# Inflammasome-Related Genetic Polymorphisms as Severity Biomarkers of COVID-19

**DOI:** 10.3390/ijms25073731

**Published:** 2024-03-27

**Authors:** Verónica Pulito-Cueto, María Sebastián Mora-Gil, Diego Ferrer-Pargada, Sara Remuzgo-Martínez, Fernanda Genre, Leticia Lera-Gómez, Pilar Alonso-Lecue, Joao Carlos Batista-Liz, Sandra Tello-Mena, Beatriz Abascal-Bolado, Sheila Izquierdo, Juan José Ruiz-Cubillán, Carlos Armiñanzas-Castillo, Ricardo Blanco, Miguel A. González-Gay, Raquel López-Mejías, José M. Cifrián

**Affiliations:** 1 Immunopathology Group, Marqués de Valdecilla University Hospital-Valdecilla Research Institute (IDIVAL), 39008 Santander, Spain; msebastian@idival.org (M.S.M.-G.); alonsolecue@hotmail.com (P.A.-L.); jbatista@idival.org (J.C.B.-L.); ricardo.blanco@scsalud.es (R.B.); rlopezmejias78@gmail.com (R.L.-M.); josemanuel.cifrian@scsalud.es (J.M.C.); 2 Department of Rheumatology, Marqués de Valdecilla University Hospital, 39008 Santander, Spain; 3 Department of Pneumology, Marqués de Valdecilla University Hospital, 39008 Santander, Spain; diegoferrer85@gmail.com (D.F.-P.); sandra.tello@scsalud.es (S.T.-M.); beatriz.abascal@scsalud.es (B.A.-B.); sheila.izquierdo@scsalud.es (S.I.); juanjose.ruizc@scsalud.es (J.J.R.-C.); 4 Valdecilla Research Institute (IDIVAL), 39011 Santander, Spain; sara.r.mtz@gmail.com (S.R.-M.); fernandagenre@gmail.com (F.G.); 5 Department of Microbiology, Marqués de Valdecilla University Hospital, 39008 Santander, Spain; letizialera@hotmail.com; 6 Department of Infectious Diseases, Marqués de Valdecilla University Hospital, 39008 Santander, Spain; carlos.arminanzas@scsalud.es; 7 School of Medicine, University of Cantabria, 39011 Santander, Spain; miguelaggay@hotmail.com; 8 Department of Rheumatology, IIS-Fundación Jiménez Díaz, 28040 Madrid, Spain

**Keywords:** COVID-19 severity, inflammasome, inflammasome-related polymorphisms

## Abstract

The most critical forms of coronavirus disease 2019 (COVID-19) are associated with excessive activation of the inflammasome. Despite the COVID-19 impact on public health, we still do not fully understand the mechanisms by which the inflammatory response influences disease prognosis. Accordingly, we aimed to elucidate the role of polymorphisms in the key genes of the formation and signaling of the inflammasome as biomarkers of COVID-19 severity. For this purpose, a large and well-defined cohort of 377 COVID-19 patients with mild (n = 72), moderate (n = 84), severe (n = 100), and critical (n = 121) infections were included. A total of 24 polymorphisms located in inflammasome-related genes (*NLRP3*, *NLRC4*, *NLRP1*, *CARD8*, *CASP1*, *IL1B*, *IL18*, *NFKB1*, *ATG16L1*, and *MIF*) were genotyped in all of the patients and in the 192 healthy controls (HCs) (who were without COVID-19 at the time of and before the study) by RT-qPCR. Our results showed that patients with mild, moderate, severe, and critical COVID-19 presented similar allelic and genotypic distribution in all the variants studied. No statistically significant differences in the haplotypic distribution of *NLRP3*, *NLRC4*, *NLRP1*, *CARD8*, *CASP1*, *IL1B*, and *ATG16L1* were observed between COVID-19 patients, who were stratified by disease severity. Each stratified group of patients presented a similar genetic distribution to the HCs. In conclusion, our results suggest that the inflammasome polymorphisms studied are not associated with the worsening of COVID-19.

## 1. Introduction

Severe acute respiratory syndrome coronavirus 2 (SARS-CoV-2) is a contagious respiratory virus responsible for the coronavirus disease 2019 (COVID-19) pandemic, which has posed a serious threat to public health [1,2]. Most patients with COVID-19 are asymptomatic or have symptoms that are usually mild, but this virus can also cause life-threatening pneumonia with significant lung inflammation, acute respiratory distress syndrome, and cardiac and renal involvement [1,2]. Commonly, mild cases of COVID-19 can be treated using over-the-counter medications, but patients with severe or critical COVID-19 have a high risk of life-threatening complications and death [1,2]. 

Accordingly, elucidating on the predictors for disease exacerbation is currently the main focus of SARS-CoV-2 research. Several studies have shown that personalized vaccination is an important consideration in the search for predictors. The waning of immunity after COVID-19 vaccination has been described as creating a constant need for boosters [3,4]. In this sense, predicting individual responses to booster vaccines may help in their timely administration. Thus, a recent large prospective study addressed this issue and showed that the waning of immunity after vaccination varies between individuals; as such, the characteristics of the humoral response to COVID-19 vaccines could be good predictors of the humoral response generated by booster vaccines [3].

Despite advances in understanding the predictors of the vaccine humoral response, the biological predictors of COVID-19 exacerbation have still not been fully defined. In this context, the aggravation of COVID-19 has a relevant relationship with the overproduction of proinflammatory cytokines, as well as with the recruitment of proinflammatory macrophages and granulocytes that cause the so-called “cytokine storm” [5,6,7,8,9,10,11]. This “cytokine storm” is frequently triggered by the activation of inflammasomes; protein complexes formed in the cytosol in response to several stimuli, which exert their function through an innate immune receptor; an adaptor protein (an adapter apoptosis-associated, speck-like protein containing a caspase recruitment domain (CARD)); and an effector enzyme (caspase-1 (CASP-1)) [5,6,7,8,9]. Several inflammasomes have been involved in viral infections, some of the most relevant being the nucleotide oligomerization domain leucine-rich repeat (NLR) pyrin-domain containing protein 3 (NLRP3), the NLR pyrin-domain containing protein 1 (NLRP1), or the NLR Family CARD containing 4 (NLRC4). When an inflammasome is stimulated, an assembly is induced and recruits CASP-1, which converts pro-interleukin (IL)-1β and pro-IL-18 to their active forms. Consequently, these cytokines trigger the inflammatory response during the viral infection [5,6,7,8,9,12]. This biological process is tightly controlled by several factors such as macrophage migration inhibitory factor (MIF) and the autophagy-related 16-like 1 (ATG16L1), which have recently been highlighted by their important role in regulating NLRP3 inflammasome activation, as well as by the release of IL-1b and IL-18 [13,14].

Interestingly, studies have proven that SARS-CoV-2 represents a pathogen-associated molecular pattern that is able to trigger inflammasome activation, whereby the over-exuberant inflammasome response has emerged as a relevant predisposing factor for disease severity and poor clinical outcome in COVID-19 patients [5,6,7,8,9]. In particular, NLRP3 and NLRP1 inflammasome activation—as well as the components of its signaling, i.e., CASP1, IL-1β, and IL-18, etc.—show a relationship with the inflammatory factors and disease progression in patients with COVID-19 [5,6,7,8,9,15,16,17,18]. In fact, some inflammasome inhibitors that exist for other non-infectious diseases have the potential to be used to treat severe SARS-CoV-2 complications [7].

Given the evident participation of the inflammasome in SARS-CoV-2 infection, it is plausible to think that inflammasome-related genes could provide new perspectives for illuminating the pathological processes potentially influencing the worsening and increased mortality of patients with this devastating disease. In fact, previous reports have suggested that the genetic variants related to the inflammasome are involved in the susceptibility or progression of COVID-19 disease [19,20,21,22,23,24,25]. However, our understanding of the complex interconnection between SARS-CoV-2 and inflammasome-related genes is still incomplete.

Accordingly, we aimed to elucidate on the role of polymorphisms located in key genes in the formation and signaling of the inflammasome (*NLRP3*, *NLRC4*, *NLRP1*, *CARD8*, *CASP1*, *IL1B*, *IL18*, *NFKB1*, *ATG16L1*, and *MIF*) as biomarkers of COVID-19 severity, which was achieved via evaluating a large and well-defined cohort of 377 patients with SARS-CoV-2 infection.

## 2. Results

### 2.1. Genotyping Quality Control

All of the genotyping results underwent rigorous quality control to ensure reliability.

The genotyping success rate was greater than 97% for all the polymorphisms evaluated. 

No deviation from HWE was detected in any of the polymorphisms at the 5% significance level in the HCs. 

### 2.2. Role of the Inflammasome-Related Polymorphisms in the Severity of COVID-19

The patients with SARS-CoV-2 infection were stratified according to the severity of the disease into mild, moderate, severe, and critical patients. They were then compared separately between each two groups on the basis of the genetic distribution of the SNPs studied in order to elucidate the influence of inflammasome-related polymorphisms on the outcome of the disease. Additionally, to confirm the results, we compared patients with mild and moderate COVID-19 together versus those with severe and critical COVID-19 together.

In a further step, we compared the whole cohort of COVID-19 patients with the HC group, which comprised individuals without COVID-19 at the time and before the study.

#### 2.2.1. Allelic and Genotypic Frequencies of Inflammasome-Related Polymorphisms in the Worsening of COVID-19

Patients with mild, moderate, severe, and critical COVID-19 showed similar allelic and genotypic frequencies in the SNPs studied in *NLRP3*, *NLRC4*, *NLRP1*, *CARD8*, *CASP1*, *IL1B*, *IL18*, *NFKB1*, *ATG16L1*, and *MIF* (see Table 1, Table 2, Table 3 and Table 4 and Supplementary Tables S1–S10—Supplementary Materials). These results were confirmed by the lack of differences when we compared patients with mild and moderate COVID-19 together versus those with severe and critical COVID-19 together.

In a further step, no statistically significant differences in the genotypic and allelic frequencies of each polymorphism studied in *NLRP3*, *NLRC4*, *NLRP1*, *CARD8*, *CASP1*, *IL1B*, *IL18*, *NFKB1*, *ATG16L1*, and *MIF* were disclosed when COVID-19 patients with mild, moderate, severe, and critical infection were compared to the HCs (see Table 1, Table 2, Table 3 and Table 4 and Supplementary Tables S11–S14—Supplementary Materials). 

#### 2.2.2. Haplotypic Frequencies of Inflammasome-Related Polymorphisms in the Worsening of COVID-19

No statistically significant differences in the haplotypic distribution of *NLRP3*, *NLRC4*, *NLRP1*, *CARD8*, *CASP1*, *IL1B*, and *ATG16L1*, with a frequency greater than 5% in the HCs, were observed between the COVID-19 patients with mild, moderate, severe, and critical infection (see Table 5 and Supplementary Table S15—Supplementary Materials). These results were confirmed by the lack of differences when we compared patients with mild and moderate COVID-19 together versus those with severe and critical COVID-19 together.

Additionally, the distribution of the haplotypes of NLRP3, NLRC4, NLRP1, CARD8, CASP1, IL1B, and ATG16L1, with a frequency greater than 5% in HC, were similar between COVID-19 patients with mild, moderate, severe, and critical infection, and HC (Table 5 and See Supplementary Table S16—Supplementary Materials).

### 2.3. Differences in the Demographic and Clinical Features of COVID-19 Patients

The patients with SARS-CoV-2 infection were stratified according to the severity of the disease into mild, moderate, severe, and critical patients. They were then compared separately as two groups with respect to demographic and clinical features. In this sense, statistically significant differences were found in the following demographic and clinical features: age, sex, arterial hypertension, diabetes mellitus type 2, previous lung disease, vaccination, D-dimer on admission, peak ferritin on admission, peak lactate dehydrogenase on admission, peak C-reactive protein on admission, corticosteroids, tocilizumab, high-flow nasal therapy, orotracheal intubation, and days of hospital admission (Table 6).

## 3. Discussion

COVID-19 exhibits a wide spectrum of clinical presentations, ranging from asymptomatic cases to severe pneumonia or even death [1,2]. In serious cases of COVID-19, a “cytokine storm” can be observed, which is triggered by excessive and uncontrolled activation of the inflammasome, thereby alluding to the crucial involvement of the inflammasome in the exacerbation of this devasting disease [5,6,7,8,9,22]. Despite the impact of COVID-19 on public health, we still do not fully understand how the inflammatory response influences disease prognosis. Accordingly, this study aimed to evaluate the role of key polymorphisms in inflammasome-related genes (*NLRP3*, *NLRC4*, *NLRP1*, *CARD8*, *CASP1*, *IL1B*, *IL18*, *NFKB1*, *ATG16L1*, and *MIF*) as risk factors of progression to critical SARS-CoV-2 infection outcomes. 

NLRP3 is the most extensively studied inflammasome in COVID-19 owing to its critical role in mediating infection-induced inflammation [5,6,7,8,9]. Interestingly, our study revealed that *NLRP3* polymorphisms do not seem to be associated with the development of more severe SARS-CoV-2 virus infections. In keeping with our results, previous findings have also disclosed that rs10754558 *NLRP3* is not associated with the worsening of COVID-19 [20]. Some studies have reported that SARS-CoV-2 infection is linked with other inflammasomes such as NLRP1 [5,6,7,8,9,16]. Therefore, we evaluated *NLRP1* polymorphisms, and we did not find genetic distribution differences between patients with mild, moderate, severe, and critical COVID-19. In this sense, only one work has described a difference of rs12150220 *NLRP1* between severe and mild Brazilian COVID-19 patients [26]. Additionally—and to the best of our knowledge—we assessed, for the first time, the potential relationship between *NLRC4* polymorphisms and the severity of COVID-19 by observing the same results as the other inflammasomes. As can be concluded from the data presented above, no genetic implication of the most important inflammasome receptors in COVID-19 aggravation was shown in our cohort of patients. 

Besides that, it is well known that inflammasome activation and signaling involve factors that have been evidenced as critical in the pathogenesis of COVID-19 [5,6,7,8,9,15,16,17,18]. In the current study, we found no association of *CARD8*, *IL1B*, and *NFKB1* polymorphisms with the poor outcome of our cohort of COVID-19 patients. This study confirms previous reports describing the same results in relation to rs6509365 *CARD8* [20], rs1143634 *IL1B* [20,21], rs16944 *IL1B* [24], and rs28362491 *NFKB1* [27]. Regarding *IL18* and *CASP1*, to the best of our knowledge, a relationship of their polymorphisms with COVID-19 exacerbation has not been previously reported. Given their importance in inflammasome signaling [5,6,7,8,9,15,16,17,18], we analyzed it for the first time, and no significant difference was found between patients with mild, moderate, severe, and critical SARS-CoV-2 virus infection. Furthermore, it should be noted that, in recent studies, ATG16L and MIF have been proposed as critical components in the regulation of inflammasome activation [13,14]. Regarding COVID-19 worsening, it seems that *ATG16L* and *MIF* polymorphisms do not play a role since our study showed that patients with different disease severity presented similar genetic distribution. Following the same line of evidence, previous findings revealed that rs2241880 *ATG16L1* [28] and rs755622 *MIF* [29] have no relationship with COVID-19 outcome. In summary, no association of inflammasome activation, signaling, and regulation genes with the worsening of our COVID-19 patients was discovered. 

It is important to mention that the previous results in the literature are controversial since other works have shown that some of the genetic variants that were studied in this work might modulate inflammasome activation, thus contributing to a worse or better prognosis in disease progression to severe outcomes. In particular, a relationship between COVID-19 aggravation and rs10754558 *NLRP3* [21,30], rs6509365 *CARD8* [21], rs2043211 *CARD8* [26], rs16944 *IL1B* [31], and rs755622 *MIF* [32] have been described. It is reasonable to think that both the difference in ethnicity and criteria for stratifying patients according to disease severity between studies are contributing to this apparent discrepancy in the data. Further validation is needed to clarify this question.

In a further step, we evaluated the differences between the whole cohort of COVID-19 patients and the HCs (who did not present COVID-19 at the time of and before the study). In this sense, no differences in genetic distribution were found between COVID-19 patients and the HCs; this finding could support the notion that these inflammasome-related genes are not involved in the COVID-19 pathogenesis. 

As a whole, our findings showed that the polymorphisms studied in the inflammasome-related genes did not seem to contribute to the worsening of SARS-CoV-2 infection in our large cohort of Caucasian patients with COVID-19. Accordingly, we could hypothesize that the abnormal hyperactivation of the inflammasome in severe COVID-19 may not be as a consequence of an alteration in the DNA sequence of the inflammasome genes, but rather due to the modifications at the transcriptional and/or translational level, which could explain the involvement of the inflammasome proteins previously evidenced in COVID-19 [5,6,7,8,9,15,16,17,18]. 

It is worth mentioning that the main limitation of our study is the differences in the baseline characteristics between the COVID-19 patient groups, which could affect the severity of the disease. However, we addressed this limitation by using changing baseline characteristics in our patient groups as confounding factors in the genetic analyses, thus reducing their potential influence on disease severity. Another limitation is the possibility that individuals in the healthy control group could develop COVID-19 after the study, so these results should be interpreted with caution.

## 4. Materials and Methods 

### 4.1. Study Population

The present study involved 377 COVID-19 patients with mild (n = 72), moderate (n = 84), severe (n = 100), and critical (n = 121) infection by the SARS-CoV-2 virus. All of the patients fulfilled the criteria for the diagnosis and classification of COVID-19 that has been established by the World Health Organization [33]. Thus, patients with a mild SARS-CoV-2 virus infection were those confirmed by laboratory analysis and were without pneumonia, whereas patients with moderate infection were those confirmed in the laboratory and who developed pneumonia. Patients with severe SARS-CoV-2 virus infection were those who manifested dyspnea, a respiratory rate of ≥30/minute, a blood oxygen saturation of ≤93%, a PaO_2_/FiO_2_ ratio of <300, and/or pulmonary infiltrates of >50% of the lung field within 24–48 h. Patients with critical infection were those who manifested severe respiratory failure and required mechanical ventilation, or those who had organ failure and required admission to an intensive care unit. All of these patients were recruited at the Pneumology Department of Hospital Universitario Marqués de Valdecilla in collaboration with the Rheumatology, Infectious Diseases, and Internal Medicine Departments of the aforementioned hospital. 

In a further step, 192 unrelated individuals without COVID-19 at the time of and before the study and without a history of pulmonary disease were enrolled in this work as healthy controls (HCs). All these individuals were ethnically and sex-matched with COVID-19 patients, and their recruitment was recorded in the National DNA Bank Repository (Salamanca).

Peripheral venous blood samples were collected from all patients and healthy individuals.

Demographic and clinical features were collected from all COVID-19 patients (Table 7). 

All the experiments involving humans and human blood samples were carried out in agreement with the approved guidelines and regulations, as well as in accordance with the guidelines detailed in the Declaration of Helsinki. All experimental protocols were approved by the Ethics Committee of Clinical Research of Cantabria, Spain. All subjects gave written informed consent to participate in this study before their inclusion.

### 4.2. Polymorphism Genotyping Method

The genomic deoxyribonucleic acid (DNA) from the COVID-19 patients and HCs was extracted from their peripheral blood using the REALPURE “SSS” kit (RBME04, REAL, Durviz S.L., Valencia, Spain). The quality and quantity of the extracted DNA was measured in a spectrophotometer (NanoDrop ND-1000, Wilmington, DE, USA). 

All subjects of the study were genotyped by TaqMan assays for the following single nucleotide polymorphisms (SNPs), which are located in the genes involved in the formation and signaling of the inflammasome that have previously been associated with the susceptibility and development of various immune and inflammatory diseases [34,35,36,37,38,39,40,41]: NLRP3 (rs4925659, rs10159239, rs10754558, and rs4353135); NLRC4 (rs385076 and rs479333); NLRP1 (rs4790797, rs8182352, rs878329, rs2670660, rs12150220, and rs6502867); CARD8 (rs11672725, rs6509365, and rs2043211); CASP1 (rs501192 and rs488992); IL1B (rs1143634 and rs16944); IL18 (rs187238); NFKB1 (rs28362491); ATG16L1 (rs2241880 and rs6754677); and MIF (rs755622). Negative controls and duplicate samples were included to check the accuracy of the genotyping. Genotyping was performed in a QuantStudio^TM^ 7 Flex real-time polymerase chain reaction (qPCR) system (Applied Biosystems, Foster City, CA, USA). The genotyping success rate for all the genetic variants included in this study was tested. Also, all genotype data were checked for their deviation from the Hardy–Weinberg equilibrium (HWE).

### 4.3. Statistical Analyses

Categorical variables were expressed as the number of individuals (n) and percentage (%), and the continuous variables as the mean ± standard deviation and median ± interquartile range.

The differences between the demographic and clinical features between patient groups were calculated by chi-square test for categorical variables and by Student’s *t*-test for continuous variables.

All allelic and genotypic frequencies of all polymorphisms studied were analyzed and compared between COVID-19 patients stratified by severity and between each of them and HC by chi-square test. The strength of association was estimated using odds ratio (OR) and 95% confidence intervals (CI). The (n) was duplicated to estimate the allele frequencies, which was achieved by considering the fact that each individual carries two alleles of each polymorphism (one allele in each chromosome). Also, an allelic combination (haplotype) analysis was performed for the *NLRP3*, *NLRC4*, *NLRP1*, *CARD8*, *CASP1*, *IL1B*, and *ATG16L1* polymorphisms. Haplotypic frequencies were calculated via the Haploview v4.2 software (http://broad.mit.edu/mpg/haploview (accessed on 26 June 2023)), and these were compared between the groups mentioned above by chi-square test. The strength of association was estimated by OR and 95% CI. All genetic statistical analyses were adjusted for the potential confounding factors (i.e., age, sex, arterial hypertension, diabetes mellitus type 2, previous lung disease, and vaccination). The *p*-values obtained from all the genetic statistical analyses were corrected using the Benjamini–Hochberg procedure (BH) for a False Discovery Rate (FDR) of 5%. Statistically significant differences after FDR correction were considered at a *p* of <0.05. 

Statistical analysis was performed using STATA statistical software 12/SE (Stata Corp., College Station, TX, USA). 

## 5. Conclusions

Our results suggest that inflammasome-related polymorphisms are not associated with the worsening and critical clinical course of COVID-19; thus, they are not considered useful biomarkers of COVID-19 severity. This study provides new insights that contribute to the discussion of the influence of inflammasomes on COVID-19 outcomes. 

## Figures and Tables

**Table 1:** The *NLRP3* and *NLRC4* genotype and allele frequencies in the COVID-19 patients, who were stratified according to the severity of the disease, and in the healthy controls.

*Locus*	SNP	COVID-19 Patients				Healthy Controls (n = 192) % (n)
		Mild Patients (n = 72) % (n)	Moderate Patients (n = 84) % (n)	Severe Patients (n = 100) % (n)	Critical Patients (n = 121) % (n)	
*NLRP3*	**rs4925659**					
	**GG**	25.35 (18)	45.24 (38)	38.38 (38)	40.00 (48)	38.62 (73)
	**GA**	53.52 (38)	39.29 (33)	45.45 (45)	44.17 (53)	49.21 (93)
	**AA**	21.13 (15)	15.48 (13)	16.16 (16)	15.83 (19)	12.17 (23)
	**G**	52.11 (74)	64.88 (109)	61.11 (121)	62.08 (149)	63.23 (239)
	**A**	47.89 (68)	35.12 (59)	77 (38.89)	37.92 (91)	36.77 (139)
	**rs10159239**					
	**AA**	36.11 (26)	30.95 (26)	22.92 (22)	21.01 (25)	22.51 (52)
	**AG**	51.39 (37)	35.71 (30)	54.17 (52)	56.30 (67)	50.26 (95)
	**GG**	12.50 (9)	33.33 (28)	22.92 (22)	22.69 (27)	22.22 (42)
	**A**	61.81 (89)	48.81 (82)	50.00 (96)	49.16 (117)	52.65 (199)
	**G**	38.19 (55)	51.19 (86)	50.00 (96)	50.84 (121)	47.35 (179)
	**rs10754558**					
	**CC**	45.71 (32)	32.53 (27)	25.00 (25)	25.00 (30)	30.21 (58)
	**CG**	42.86 (30)	33.73 (28)	56.00 (56)	55.83 (67)	50.00 (96)
	**GG**	11.43 (8)	33.73 (28)	19.00 (19)	19.17 (23)	18.79 (38)
	**C**	67.14 (96)	49.40 (82)	53.00 (106)	52.92 (127)	55.21 (212)
	**G**	32.86 (46)	50.60 (84)	47.00 (94)	47.08 (113)	44.79 (172)
	**rs4353135**					
	**TT**	45.71 (32)	29.27 (24)	29.47 (28)	37.29 (44)	34.22 (64)
	**TG**	40.00 (28)	56.10 (46)	55.79 (53)	42.37 (50)	51.34 (96)
	**GG**	14.29 (10)	14.63 (12)	14.74 (14)	20.34 (24)	14.44 (27)
	**T**	65.71 (92)	57.32 (94)	57.37 (109)	58.47 (138)	59.89 (224)
	**G**	34.29 (48)	42.68 (70)	42.63 (81)	41.53 (98)	40.11 (150)
*NLRC4*	**rs385076**					
	**CC**	38.89 (28)	38.10 (32)	33.00 (33)	46.28 (56)	34.04 (64)
	**CT**	50.00 (36)	42.86 (36)	53.00 (53)	43.80 (53)	51.60 (97)
	**TT**	11.11 (8)	19.05 (16)	14.00 (14)	9.92 (12)	14.36 (27)
	**C**	64.34 (92)	59.52 (100)	59.50 (119)	68.18 (165)	59.84 (225)
	**T**	35.66 (51)	40.48 (68)	40.50 (81)	31.82 (77)	40.16 (151)
	**rs479333**					
	**GG**	34.72 (25)	35.80 (29)	30.21 (29)	42.86 (51)	31.25 (60)
	**GC**	51.39 (37)	40.74 (33)	54.17 (52)	42.86 (51)	52.60 (101)
	**CC**	13.89 (10)	23.46 (19)	15.63 (15)	14.29 (17)	16.15 (31)
	**G**	60.84 (87)	56.17 (91)	57.29 (110)	64.29 (153)	57.55 (221)
	**C**	39.16 (56)	43.83 (71)	42.71 (82)	35.71 (85)	42.45 (163)

COVID-19: coronavirus 19 disease. (n) represents the number of individuals successfully genotyped. The (n) was duplicated to estimate the allele frequencies, which was achieved via considering the fact that each individual carries two alleles of each polymorphism (one allele in each chromosome).

**Table 2:** The *NLRP1* genotype and allele frequencies in the COVID-19 patients, who were stratified according to the severity of the disease, and in the healthy controls.

*Locus*	SNP	COVID-19 Patients				Healthy Controls (n = 192) % (n)
		Mild Patients (n = 72) % (n)	Moderate Patients (n = 84) % (n)	Severe Patients (n = 100) % (n)	Critical Patients (n = 121) % (n)	
*NLRP1*	**rs4790797**					
	**GG**	26.39 (19)	21.95 (18)	27.08 (26)	28.33 (34)	29.28 (53)
	**GA**	48.61 (35)	57.32 (47)	56.25 (54)	45.00 (54)	46.96 (85)
	**AA**	25.00 (18)	20.73 (17)	16.67 (16)	26.67 (32)	23.76 (43)
	**G**	50.69 (73)	50.61 (83)	55.21 (106)	50.83 (122)	52.76 (191)
	**A**	49.31 (71)	49.39 (81)	44.79 (86)	49.17 (118)	47.24 (171)
	**rs8182352**					
	**TT**	27.78 (20)	21.69 (18)	27.55 (27)	28.33 (34)	29.84 (57)
	**TC**	47.22 (34)	57.83 (48)	55.10 (54)	45.83 (55)	45.03 (86)
	**CC**	25.00 (18)	20.48 (17)	17.35 (17)	25.83 (31)	25.13 (48)
	**T**	51.39 (74)	50.60 (84)	55.10 (108)	51.25 (123)	52.36 (200)
	**C**	48.61 (70)	49.40 (82)	44.90 (88)	48.75 (117)	47.64 (182)
	**rs878329**					
	**GG**	28.17 (20)	22.62 (19)	26.80 (26)	28.57 (34)	30.53 (58)
	**GC**	45.07 (32)	57.14 (48)	56.70 (55)	44.54 (53)	43.68 (83)
	**CC**	26.76 (19)	20.24 (17)	16.49 (16)	26.89 (32)	25.79 (49)
	**G**	50.70 (72)	51.19 (86)	55.15 (107)	50.84 (121)	52.37 (199)
	**C**	49.30 (70)	48.81 (82)	44.85 (87)	49.16 (117)	47.63 (181)
	**rs2670660**					
	**AA**	30.99 (22)	26.19 (22)	25.77 (25)	24.58 (29)	31.25 (60)
	**AG**	47.89 (34)	52.38 (44)	56.70 (55)	50.00 (59)	43.75 (84)
	**GG**	21.13 (15)	21.43 (18)	17.53 (17)	25.42 (30)	25.00 (48)
	**A**	54.93 (78)	52.38 (88)	54.12 (105)	49.58 (117)	53.13 (204)
	**G**	45.07 (64)	47.62 (80)	45.88 (89)	50.42 (119)	46.88 (180)
	**rs12150220**					
	**AA**	32.86 (23)	29.76 (25)	24.47 (23)	28.33 (34)	31.58 (60)
	**AT**	52.86 (37)	48.81 (41)	57.45 (54)	48.33 (58)	44.74 (85)
	**TT**	14.29 (10)	21.43 (18)	18.09 (17)	23.33 (28)	23.68 (45)
	**A**	59.29 (83)	54.17 (91)	53.19 (100)	52.50 (126)	53.95 (205)
	**T**	40.71 (57)	45.83 (77)	46.81 (88)	47.50 (114)	46.05 (175)
	**rs6502867**					
	**TT**	55.71 (39)	48.19 (40)	52.13 (49)	52.59 (61)	61.11 (110)
	**TC**	34.29 (24)	43.37 (36)	44.68 (42)	40.52 (47)	33.89 (61)
	**CC**	10.00 (7)	8.43 (7)	3.19 (3)	6.90 (8)	5.00 (9)
	**T**	72.86 (102)	69.88 (116)	74.47 (140)	72.84 (169)	78.06 (281)
	**C**	27.14 (38)	30.12 (50)	25.53 (48)	27.16 (63)	21.94 (79)

COVID-19: coronavirus 19 disease. (n) represents the number of individuals successfully genotyped. The (n) was duplicated to estimate the allele frequencies, which was achieved via considering the fact that each individual carries two alleles of each polymorphism (one allele in each chromosome).

**Table 3:** The *CARD8* and *CASP1* genotype and allele frequencies in the COVID-19 patients, who were stratified according to the severity of the disease, and in the healthy controls.

*Locus*	SNP	COVID-19 Patients				Healthy Controls (n = 192) % (n)
		Mild Patients (n = 72) % (n)	Moderate Patients (n = 84) % (n)	Severe Patients (n = 100) % (n)	Critical Patients (n = 121) % (n)	
*CARD8*	**rs11672725**					
	**CC**	63.38 (45)	67.47 (56)	70.83 (68)	57.63 (68)	67.02 (128)
	**CT**	33.80 (24)	26.51 (22)	26.04 (25)	38.14 (45)	28.27 (54)
	**TT**	2.82 (2)	6.02 (5)	3.13 (3)	4.24 (5)	4.71 (9)
	**C**	80.14 (113)	80.72 (134)	83.85 (161)	76.69 (181)	81.15 (310)
	**T**	19.86 (28)	19.28 (32)	16.15 (31)	23.31 (55)	18.85 (72)
	**rs6509365**					
	**AA**	58.33 (42)	45.24 (38)	35.35 (35)	50.85 (60)	40.84 (78)
	**AG**	30.56 (22)	45.24 (38)	56.57 (56)	38.14 (45)	49.21 (94)
	**GG**	11.11 (8)	9.52 (8)	8.08 (8)	11.02 (13)	9.95 (19)
	**A**	74.13 (106)	67.86 (114)	63.64 (126)	69.92 (165)	65.45 (250)
	**G**	25.87 (37)	32.14 (54)	36.36 (72)	30.08 (71)	34.55 (132)
	**rs2043211**					
	**AA**	59.15 (42)	50.00 (42)	36.36 (36)	51.24 (62)	42.19 (81)
	**AT**	29.58 (21)	41.67 (35)	55.56 (55)	38.84 (47)	48.44 (93)
	**TT**	11.27 (8)	8.33 (7)	8.08 (8)	9.92 (12)	9.38 (18)
	**A**	73.94 (105)	70.83 (119)	64.14 (127)	70.66 (171)	66.41 (255)
	**T**	26.06 (37)	29.17 (49)	35.86 (71)	29.34 (71)	33.59 (129)
*CASP1*	**rs501192**					
	**CC**	60.00 (42)	74.07 (60)	66.33 (65)	62.93 (73)	65.45 (125)
	**CT**	37.14 (26)	18.52 (15)	27.55 (27)	33.62 (39)	32.46 (62)
	**TT**	2.86 (2)	7.41 (6)	6.12 (6)	3.45 (4)	2.09 (4)
	**C**	78.42 (109)	83.33 (135)	80.10 (157)	79.74 (185)	81.68 (312)
	**T**	21.58 (30)	16.67 (27)	19.90 (39)	20.26 (47)	18.32 (70)
	**rs488992**					
	**GG**	84.72 (61)	83.13 (69)	85.00 (85)	79.34 (96)	75.92 (145)
	**GA**	13.89 (10)	14.46 (12)	13.00 (13)	19.01 (23)	23.04 (44)
	**AA**	1.39 (1)	2.41 (2)	2.00 (2)	1.65 (2)	1.05 (2)
	**G**	91.61 (131)	90.36 (150)	91.50 (183)	88.84 (215)	87.43 (334)
	**A**	8.39 (12)	9.64 (16)	8.50 (17)	11.16 (27)	12.57 (48)

COVID-19: coronavirus 19 disease. (n) represents the number of individuals successfully genotyped. The (n) was duplicated to estimate the allele frequencies, which was achieved via considering the fact that each individual carries two alleles of each polymorphism (one allele in each chromosome).

**Table 4:** The *IL1β*, *IL18*, *NFKB1*, *ATG16L1*, and *MIF* genotype and allele frequencies in the COVID-19 patients, who were stratified according to the severity of the disease, and in the healthy controls.

*Locus*	SNP	COVID-19 Patients				Healthy Controls (n = 192) % (n)
		Mild Patients (n = 72) % (n)	Moderate Patients (n = 84) % (n)	Severe Patients (n = 100) % (n)	Critical Patients (n = 121) % (n)	
*IL1B*	**rs1143634**					
	**GG**	62.50 (45)	63.10 (53)	61.00 (61)	60.00 (72)	59.34 (108)
	**GA**	33.33 (24)	34.52 (29)	31.00 (34)	36.67 (44)	33.52 (61)
	**AA**	4.17 (3)	2.38 (2)	5.00 (5)	3.33 (4)	7.14 (13)
	**G**	79.17 (114)	80.36 (135)	78.00 (156)	78.33 (188)	76.10 (277)
	**A**	20.83 (30)	19.64 (33)	22.00 (44)	21.67 (52)	23.90 (87)
	**rs16944**					
	**GG**	50.00 (36)	46.43 (39)	47.00 (47)	45.00 (54)	44.25 (77)
	**GA**	41.67 (30)	36.90 (31)	44.00 (44)	42.50 (51)	45.98 (80)
	**AA**	8.33 (6)	16.67 (14)	9.00 (9)	12.50 (15)	9.77 (17)
	**G**	70.83 (102)	64.88 (109)	69.00 (138)	66.25 (159)	67.24 (234)
	**A**	29.17 (42)	35.12 (59)	31.00 (62)	33.75 (81)	32.76 (114)
*IL18*	**rs187238**					
	**CC**	50.00 (35)	51.81 (43)	65.66 (65)	57.39 (66)	52.46 (96)
	**CG**	42.86 (30)	37.35 (31)	25.25 (25)	36.52 (42)	38.80 (71)
	**GG**	7.14 (5)	10.84 (9)	9.09 (9)	6.09 (7)	8.74 (16)
	**C**	71.43 (100)	70.48 (117)	78.28 (155)	75.65 (174)	71.86 (263)
	**G**	28.57 (40)	29.52 (49)	21.72 (43)	24.35 (56)	28.14 (103)
*NFKB1*	**rs28362491**					
	**ATTGATTG/ATTGATTG**	35.21 (25)	40.48 (34)	39.39 (39)	48.76 (59)	37.50 (72)
	**ATTGATTG/ATTG**	53.52 (38)	53.57 (45)	46.46 (46)	38.84 (47)	51.04 (98)
	**ATTG/ATTG**	11.27 (8)	5.95 (5)	14.14 (14)	12.40 (15)	11.46 (22)
	**ATTGATTG**	61.70 (87)	67.26 (113)	62.63 (124)	68.18 (165)	63.02 (242)
	**ATTG**	38.30 (54)	32.74 (55)	37.37 (74)	31.82 (77)	36.98 (142)
*ATG16L1*	**rs2241880**					
	**GG**	20.83 (15)	25.30 (21)	28.00 (28)	29.75 (36)	30.51 (54)
	**GA**	51.39 (37)	46.99 (39)	51.00 (51)	48.76 (59)	51.41 (91)
	**AA**	27.78 (20)	27.71 (23)	21.00 (21)	21.49 (26)	18.08 (32)
	**G**	46.15 (66)	48.80 (81)	53.50 (107)	54.13 (131)	56.21 (199)
	**A**	53.85 (77)	51.20 (85)	46.50 (93)	45.87 (111)	43.79 (155)
	**rs6754677**					
	**AA**	32.39 (23)	35.71 (30)	45.00 (45)	41.32 (50)	40.44 (74)
	**AG**	47.89 (34)	48.81 (41)	44.00 (44)	47.11 (57)	47.54 (87)
	**GG**	19.72 (14)	15.48 (13)	11.00 (11)	11.57 (14)	12.02 (22)
	**A**	56.03 (79)	60.12 (101)	67.00 (134)	64.88 (157)	64.21 (235)
	**G**	43.97 (62)	39.88 (67)	33.00 (66)	35.12 (85)	35.79 (131)
*MIF*	**rs755622**					
	**GG**	65.28 (47)	75.00 (63)	69.70 (69)	72.50 (87)	71.88 (138)
	**GC**	31.94 (23)	22.62 (19)	29.29 (29)	24.17 (29)	24.48 (47)
	**CC**	2.78 (2)	2.38 (2)	1.01 (1)	3.33 (4)	3.65 (7)
	**G**	81.82 (117)	86.31 (145)	84.34 (167)	84.58 (203)	84.11 (323)
	**C**	18.18 (26)	13.69 (23)	15.66 (31)	15.42 (37)	15.89 (61)

COVID-19: coronavirus 19 disease. (n) represents the number of individuals successfully genotyped. The (n) was duplicated to estimate the allele frequencies, which was achieved via considering the fact that each individual carries two alleles of each polymorphism (one allele in each chromosome).

**Table 5:** The *NLRP3*, *NLRC4*, *NLRP1*, *CARD8*, *CASP1*, *IL1β*, and *ATG16L1* haplotype frequencies in COVID-19 patients, who were stratified according to the severity of the disease, and in the healthy controls.

*Locus*	Haplotypes	COVID-19 Patients				Healthy Controls (n = 192) % (n)
		Mild Patients (n = 72) % (n)	Moderate Patients (n = 84) % (n)	Severe Patients (n = 100) % (n)	Critical Patients (n = 121) % (n)	
*NLRP3 * ^1^	**AACT**	38.00 (55)	29.30 (49)	29.30 (59)	26.60 (64)	25.50 (98)
	**GGGT**	15.00 (22)	19.20 (32)	19.10 (38)	22.30 (54)	24.20 (93)
	**GGGG**	17.30 (25)	30.80 (52)	27.30 (55)	24.80 (60)	20.60 (79)
	**AACG**	9.00 (13)	5.80 (10)	9.40 (19)	11.40 (28)	11.10 (43)
	**GACG**	8.20 (12)	6.30 (11)	6.10 (12)	5.40 (13)	8.40 (32)
	**GACT**	5.80 (8)	7.40 (12)	5.10 (10)	6.20 (15)	7.30 (28)
*NLRC4 * ^2^	**CG**	58.90 (85)	56.40 (95)	57.30 (115)	63.90 (155)	57.60 (141)
	**TC**	34.60 (50)	40.50 (68)	39.50 (79)	31.00 (75)	40.60 (96)
*NLRP1 * ^3^	**TTGCCA**	34.70 (50)	36.50 (61)	36.60 (73)	38.00 (92)	39.10 (150)
	**TAAGTG**	28.30 (41)	26.70 (45)	30.80 (62)	26.30 (64)	31.60 (121)
	**CAAGTG**	18.10 (26)	19.40 (33)	18.90 (38)	17.20 (42)	17.00 (65)
*CARD8 * ^4^	**CAA**	56.40 (81)	50.90 (86)	47.50 (95)	49.20 (119)	49.10 (189)
	**CGT**	22.50 (33)	28.00 (47)	35.00 (70)	27.00 (65)	31.70 (122)
	**TAA**	15.90 (23)	16.90 (29)	16.00 (32)	21.10 (51)	16.50 (63)
*CASP1 * ^5^	**CG**	78.80 (113)	83.70 (141)	80.30 (161)	80.20 (194)	81.50 (313)
	**TA**	8.30 (12)	9.50 (16)	8.5 (17)	11.20 (27)	12.40 (48)
	**TG**	12.90 (19)	6.80 (12)	11.20 (23)	8.70 (21)	5.90 (23)
*IL1B * ^6^	**GG**	50.00 (72)	47.90 (79)	49.70 (99)	48.00 (116)	47.90 (176)
	**AG**	29.20 (42)	33.10 (56)	28.30 (57)	30.40 (74)	28.20 (104)
	**GA**	20.80 (30)	17.60 (30)	19.30 (39)	18.30 (44)	19.30 (71)
*ATG16L1 * ^7^	**GA**	44.10 (64)	45.70 (77)	50.50 (101)	53.20 (129)	52.10 (195)
	**AG**	41.20 (59)	36.50 (61)	30.00 (60)	34.20 (83)	32.00 (120)
	**AA**	12.30 (18)	14.40 (24)	16.50 (33)	11.70 (28)	12.20 (46)

COVID-19: coronavirus 19 disease. The order of the polymorphisms was as follows: ^1^ rs4925659, rs10159239, rs10754558, and rs4353135 for *NLRP3*; ^2^ rs385076 and rs479333 for *NLRC4;* ^3^ rs4790797, rs8182352, rs878329, rs2670660, rs12150220, and rs6502867 for *NLRP1*; ^4^ rs11672725, rs6509365, and rs2043211 for *CARD8*; ^5^ rs501192 and rs488992 for *CASP1;* ^6^ rs1143634 and rs16944 for *IL1B*; ^7^ and rs2241880 and rs6754677 for *ATG16L1*. Haplotypes with a frequency higher than 5% in the healthy controls are displayed in the table.

**Table 6:** Differences in the demographic and clinical features between COVID-19 mild patients, moderate patients, and severe patients, as well as critical patients.

Features	COVID-19 Patients					
	Mild vs. Moderate *p*	Mild vs. Severe *p*	Mild vs. Critical *p*	Moderate vs. Severe *p*	Moderate vs. *Critical* *p*	Severe vs. *Critical* *p*
Age	0.58	**<0.01**	**<0.01**	**<0.01**	**<0.01**	0.64
Male sex	0.04	**<0.01**	**<0.01**	0.09	**<0.01**	0.28
Smoking	0.61	0.38	0.41	0.65	0.68	0.93
Body mass index	0.82	0.79	0.84	0.99	0.86	0.78
Arterial hypertension	0.11	**<0.01**	**<0.01**	0.15	**<0.01**	0.16
Diabetes mellitus type 2	0.80	**0.02**	0.08	**0.03**	0.11	0.49
Ischemic heart disease	0.55	0.21	0.61	0.06	0.25	0.36
Chronic kidney disease	0.91	0.20	0.14	0.15	0.09	0.80
Onco-hematological pathology	0.77	0.67	0.63	0.88	0.84	0.96
Previous lung disease	0.07	0.15	0.65	0.65	**<0.01**	**0.03**
Immunosuppression	0.34	0.22	0.63	0.77	0.55	0.35
Vaccination	**0.04**	0.56	0.84	0.12	**0.02**	0.37
D-dimer on admission (ng/mL)	-	-	**-**	0.54	**<0.01**	**<0.01**
Peak ferritin on admission (ng/mL)	-	-	-	**0.02**	**<0.01**	**0.01**
Peak LDH on admission (U/L)	-	-	-	**<0.01**	**<0.01**	**<0.01**
Peak CRP on admission (mg/dL)	-	-	-	**<0.01**	**<0.01**	**<0.01**
Corticosteroids	-	-	-	**<0.01**	**<0.01**	**<0.01**
Tocilizumab	-	-	-	**<0.01**	**<0.01**	**<0.01**
High flow nasal therapy	-	-	-	0.68	**<0.01**	**<0.01**
Orotracheal intubation	-	-	-	-	**<0.01**	**<0.01**
Days of hospital admission	-	-	-	0.41	**<0.01**	**<0.01**

COVID-19: coronavirus 19 disease; LDH: lactate dehydrogenase; and CRP: C-reactive protein. *p*-values of < 0.05 are highlighted in **bold**.

**Table 7:** The demographic and clinical features of COVID-19 patients, who were stratified according to the severity of the disease.

*Demographic and Clinical Features*	COVID-19 Patients			
	Mild Patients (n = 72)	Moderate Patients (n = 84)	Severe Patients (n = 100)	Critical Patients (n = 121)
Age, mean ± SD	55.65 ± 1.43	57.86 ± 1.42	65.37 ± 1.18	63.8 ± 1.00
Male sex, n (%)	45 (62.5)	39 (46.34)	34 (34.00)	33 (27.27)
Smoking, n (%)	12 (16.66)	14 (17.07)	12 (12.00)	15 (12.39)
Body mass index, median (IQR)	28.96 (27.76–29.65)	29.53 (28.38–30.68)	29.43 (28.53–30.33)	29.85 (29.10–30.60)
Arterial hypertension, n (%)	14 (19.44)	27 (32.14)	45 (45.00)	66 (54.55)
Diabetes mellitus type 2, n (%)	6 (8.33)	8 (9.75)	21 (21.00)	21 (17.35)
Ischemic heart disease, n (%)	4 (5.50)	3 (3.65)	11 (11.00)	9 (7.48)
Chronic kidney disease, n (%)	1 (1.38)	1 (1.01)	5 (5.00)	7 (5.78)
Onco-hematological pathology, n (%)	2 (2.77)	3 (3.65)	4 (4.00)	5 (4.13)
Previous lung disease, n (%)	4 (5.56)	12 (14.29)	12 (12.00)	5 (4.13)
Immunosuppression, n (%)	2 (2.77)	5 (6.09)	7 (7.00)	5 (4.13)
Vaccination, n (%)	14 (19.44)	7 (8.33)	16 (16.00)	25 (20.66)
One dose, n (%)	8 (11.11)	4 (4.76)	7 (7.00)	12 (9.92)
Two doses, n (%)	6 (8.33)	3 (3.57)	9 (9.00)	13 (10.74)
D-dimer on admission (ng/mL), median (IQR)	-	1860.90 (244.69–3477.11)	3669.02 (1964.49–5373.55)	8582.14 (6039.26–11125.02)
Peak ferritin on admission (ng/mL), median (IQR)	-	576.96 (450.82–703.10)	976.90 (799.5–1154.30)	1331.40 (1128.41–1534.39)
Peak LDH on admission (U/L), median (IQR)	-	271.87 (254.20–289.55)	358.97 (330.41–387.52)	448.22 (417.84–478.60)
Peak CRP on admission (mg/dL), median (IQR)	-	10.78 (8.56–12.67)	12.20 (10.69–13.72)	16.85 (15.40–18.41)
Corticosteroids, n (%)	-	12 (14.63)	63 (63.00)	107 (88.42)
Tocilizumab, n (%)	-	0 (0.00)	18 (18.00)	41 (33.88)
High flow nasal therapy, n (%)	-	0 (0.00)	3 (3.00)	88 (72.72)
Orotracheal intubation, n (%)	-	0 (0.00)	0 (0.00)	83 (68.59)
Days of hospital admission, median (IQR)	-	7.89 (6.83–8.94)	10.52 (9.69–11.36)	25.93 (22.54–29.32)

COVID-19: coronavirus 19 disease; IQR: interquartile range; LDH: lactate dehydrogenase; and CRP: C-reactive protein.

## Data Availability

All data generated or analyzed during this study are included in this published article [and its Supplementary Information Files].

## Supplementary Materials

The supporting information can be downloaded at: https://www.mdpi.com/article/10.3390/ijms25073731/s1.
