# Supplementary material for: Inflammasome-Related Genetic Polymorphisms as Severity Biomarkers of COVID-19
Source: Int J Mol Sci. 2024 Mar 27;25(7):3731. doi: 10.3390/ijms25073731 (PMC11011752; doi:10.3390/ijms25073731)
Supplement: Supplementary file 1 [file ijms-25-03731-s001.zip › ijms-2896142-supplementary.pdf]

Additional Files

Inflammasome-related genetic polymorphisms as severity biomarkers of COVID-19?

Verónica Pulito-Cueto[1,2*¥], María Sebastián Mora-Gil[1,2*], Diego Ferrer[3], Sara Remuzgo-Martínez[4], Fernanda Genre[4], Leticia Lera-Gómez[5], Pilar Alonso-Lecue[1,3], Joao Carlos Batista-Liz[1,2], Sandra Tello-Mena[3], Beatriz Abascal-Bolado[3], Sheila Izquierdo[3], Juan José Ruiz-Cubillan[3], Cárlos Armiñanzas-Castillo[6], Ricardo Blanco[1,2], Miguel A. González-Gay[7,8], Raquel López-Mejías[1,2**], and José M. Cifrián[1,3,7**]

Table S1. Differences in *NLRP3* genotype and allele distribution between COVID-19 mild patients and moderate patients, severe patients as well as critical patients.

		COVID-19 Patients											
Locus	SNP	**Mild *vs* moderate**		**Mild *vs* Severe**		**Mild *vs* Critical**		**Moderate *vs* Severe**		**Moderate *vs Critical***		**Severe *vs Critical***	
		p	OR [95% CI]	*p*	OR [95% CI]	*p*	OR [95% CI]	*p*	OR [95% CI]	*p*	OR [95% CI]	*p*	OR [95% CI]
NLRP3	**rs4925659**												
	**GG**	-	Ref.	-	Ref.	-	Ref.	-	Ref.	-	Ref.	-	Ref.
	**GA**	0.02*	2.54[1.14-5.66]	0.15	1.87[0.80-4.37]	0.03*	2.55[1.11-5.85]	0.47	0.77[0.39-1.55]	0.88	0.95[0.49-1.84]	0.72	1.11[0.61-2.04]
	**AA**	0.22	1.90[0.68-5.30]	0.06	2.87[0.97-8.53]	0.05	2.85[0.99-8.18]	0.75	0.86[0.34-2.18]	0.81	1.12[0.45-2.79]	0.98	0.99[0.44-2.23]
	**G**	-	Ref.	-	Ref.	-	Ref.	-	Ref.	-	Ref.	-	Ref.
	**A**	0.09	1.53[0.93-2.52]	0.05	1.69[1.00-2.86]	0.03*	1.75[1.06-2.89]	0.61	0.89[0.56-1.40]	0.89	1.03[0.66-1.62]	0.92	1.02[0.69-1.52]
	**rs10159239**												
	**AA**	-	Ref.	-	Ref.	-	Ref.	-	Ref.	-	Ref.	-	Ref.
	**AG**	0.34	1.48[0.66-3.31]	0.14	0.52[0.22-1.23]	0.044	0.42[0.18-0.98]	0.06	0.48[0.22-1.04]	0.01*	0.36[0.17-0.79]	0.98	0.99[0.49-2.00]
	**GG**	0.05	0.36[0.13-0.98]	0.02*	0.26[0.08-0.82]	0.017	0.25[0.08-0.78]	0.60	1.27[0.52-3.10]	0.67	0.83[0.35-1.95]	1.00	1.00[0.44-2.30]
	**A**	-	Ref.	-	Ref.	-	Ref.	-	Ref.	-	Ref.	-	Ref.
	**G**	0.08	0.64[0.39-1.05]	0.02*	0.54[0.32-0.92]	0.016	0.54[0.33-0.89]	0.67	1.10[0.70-1.72]	0.74	0.93[0.60-1.43]	1.00	1.00[0.66-1.48]
	**rs10754558**												
	**CC**	-	Ref.	-	Ref.	-	Ref.	-	Ref.	-	Ref.	-	Ref.
	**CG**	0.74	0.87[0.39-1.96]	<0.01*	0.26[0.11-0.62]	<0.01*	0.32[0.14-0.71]	0.03*	0.42[0.19-0.91]	0.03*	0.44[0.21-0.94]	0.74	1.12[0.58-2.17]
	**GG**	0.01*	0.26[0.09-0.72]	0.02*	0.23[0.07-0.78]	0.03*	0.29[0.09-0.90]	0.24	1.73[0.69-4.34]	0.50	1.34[0.57-3.13]	0.79	1.12[0.49-2.60]
	**C**	-	Ref.	-	Ref.	-	Ref.	-	Ref.	-	Ref.	-	Ref.
	**G**	<0.01*	0.50[0.30-0.83]	<0.01*	0.45[0.26-0.79]	<0.01*	0.50[0.30-0.84]	0.36	1.23[0.79-1.93]	0.58	1.13[0.73-1.75]	0.79	1.06[0.72-1.55]
	**rs4353135**												
	**TT**	-	Ref.	-	Ref.	-	Ref.	-	Ref.	-	Ref.	-	Ref.
	**TG**	0.04*	0.43[0.20-0.96]	0.03*	0.39[0.17-0.89]	0.24	0.62[0.28-1.37]	0.66	0.85[0.41-1.77]	0.14	1.70[0.85-3.41]	0.12	1.66[0.88-3.14]
	**GG**	0.36	0.60[0.20-1.78]	0.41	0.62[0.20-1.94]	0.23	0.53[0.19-1.49]	0.82	0.89[0.31-2.53]	0.62	0.79[0.31-2.02]	0.78	0.89[0.39-2.03]
	**T**	-	Ref.	-	Ref.	-	Ref.	-	Ref.	-	Ref.	-	Ref.
	**G**	0.16	0.69[0.42-1.15]	0.16	0.68[0.40-1.16]	0.15	0.68[0.41-1.14]	0.77	0.94[0.60-1.47]	0.99	1.00[0.64-1.55]	0.89	1.03[0.69-1.53]

COVID-19: coronavirus 19 disease. *The statistical significance was lost after correcting for multiple testing using the Benjamini-Hochberg method for a False Discovery Rate (FDR) of 5%.

Table S2. Differences in *NLRC4* genotype and allele distribution between COVID-19 mild patients and moderate patients, severe patients as well as critical patients.

		COVID-19 Patients											
Locus	SNP	Mild *vs* moderate		Mild *vs* Severe		Mild *vs* Critical		Moderate *vs* Severe		Moderate *vs Critical*		Severe *vs Critical*	
		p	OR [95% CI]	*p*	OR [95% CI]	*p*	OR [95% CI]	*p*	OR [95% CI]	*p*	OR [95% CI]	*p*	OR [95% CI]
NLRC4	**rs385076**												
	**CC**	-	Ref.	-	Ref.	-	Ref.	-	Ref.	-	Ref.	-	Ref.
	**CT**	0.84	1.08[0.51-2.27]	0.74	0.88[0.40-1.92]	0.97	0.98[0.47-2.04]	0.40	0.74[0.37-1.48]	0.93	1.03[0.53-2.01]	0.09	1.68[0.93-3.04]
	**TT**	0.18	0.47[0.16-1.42]	0.40	0.60[0.18-1.98]	0.90	0.92[0.29-3.00]	0.66	1.23[0.49-3.12]	0.19	1.86[0.73-4.76]	0.13	2.01[0.81-5.01]
	**C**	-	Ref.	-	Ref.	-	Ref.	-	Ref.	-	Ref.	-	Ref.
	**T**	0.31	0.77[0.46-1.27]	0.44	0.81[0.48-1.38]	0.90	0.97[0.58-1.61]	0.90	1.03[0.66-1.61]	0.27	1.29[0.82-2.01]	0.07	1.45[0.97-2.17]
	**rs479333**												
	**GG**	-	Ref.	-	Ref.	-	Ref.	-	Ref.	-	Ref.	-	Ref.
	**GC**	0.55	1.27[0.59-2.73]	0.86	0.93[0.42-2.08]	0.67	1.18[0.56-2.49]	0.27	0.66[0.32-1.37]	0.88	0.95[0.47-1.90]	0.08	1.73[0.94-3.19]
	**CC**	0.54	0.72[0.26-2.01]	0.91	1.07[0.34-3.34]	0.92	1.06[0.36-3.09]	0.44	1.44[0.57-3.64]	0.31	1.55[0.66-3.67]	0.34	1.51[0.64-3.56]
	**G**	-	Ref.	-	Ref.	-	Ref.	-	Ref.	-	Ref.	-	Ref.
	**C**	0.71	0.81[0.55-1.50]	0.98	1.01[0.60-1.69]	0.83	1.06[0.64-1.74]	0.66	1.11[0.70-1.74]	0.37	1.22[0.79-1.90]	0.18	1.32[0.88-1.96]

COVID-19: coronavirus 19 disease.

Table S3. Differences in *NLRP1* genotype and allele distribution between COVID-19 mild patients and moderate patients, severe patients as well as critical patients.

Locus	SNP	COVID-19 Patients											
		**Mild *vs* moderate**		**Mild *vs* Severe**		**Mild *vs* Critical**		**Moderate *vs* Severe**		**Moderate *vs Critical***		**Severe *vs Critical***	
		p	OR [95% CI]	*p*	OR [95% CI]	*p*	OR [95% CI]	*p*	OR [95% CI]	*p*	OR [95% CI]	*p*	OR [95% CI]
NLRP1	**rs4790797**												
	**GG**	-	Ref.	-	Ref.	-	Ref.	-	Ref.	-	Ref.	-	Ref.
	**GA**	0.58	0.79[0.34-1.84]	0.63	0.90[0.33-1.95]	0.82	1.10[0.47-2.59]	0.55	1.27[0.59-2.75]	0.21	1.63[0.76-3.52]	0.53	1.24[0.64-2.37]
	**AA**	0.61	1.30[0.48-3.54]	0.29	1.77[0.61-5.13]	0.96	1.02[0.40-2.62]	0.47	1.43[0.54-3.81]	0.90	1.06[0.44-2.57]	0.30	0.66[0.30-1.46]
	**G**	-	Ref.	-	Ref.	-	Ref.	-	Ref.	-	Ref.	-	Ref.
	**A**	0.63	1.13[0.69-1.83]	0.35	1.28[0.77-2.13]	0.95	1.02[0.62-1.65]	0.49	1.17[0.75-1.83]	0.88	1.03[0.67-1.59]	0.37	0.84[0.57-1.23]
	**rs8182352**												
	**TT**	-	Ref.	-	Ref.	-	Ref.	-	Ref.	-	Ref.	-	Ref.
	**TC**	0.46	0.72[0.31-1.69]	0.67	0.82[0.34-2.00]	0.99	1.00[0.43-2.32]	0.58	1.24[0.58-2.67]	0.29	1.50[0.71-3.19]	0.70	1.14[0.59-2.17]
	**CC**	0.67	1.25[0.46-3.40]	0.37	1.61[0.57-4.60]	0.84	1.10[0.43-2.81]	0.56	1.33[0.51-3.49]	0.85	1.09[0.45-2.61]	0.36	0.69[0.32-1.53]
	**T**	-	Ref.	-	Ref.	-	Ref.	-	Ref.	-	Ref.	-	Ref.
	**C**	0.70	1.10[0.68-1.79]	0.42	1.24[0.74-2.06]	0.82	1.06[0.65-1.72]	0.58	1.14[0.73-1.77]	0.81	1.05[0.68-1.69]	0.42	0.85[0.58-1.26]
	**rs878329**												
	**GG**	-	Ref.	-	Ref.	-	Ref.	-	Ref.	-	Ref.	-	Ref.
	**GC**	0.53	0.76[0.33-1.76]	0.54	0.75[0.30-1.88]	0.89	1.06[0.45-2.50]	0.72	1.15[0.53-2.49]	0.24	1.58[0.74-3.36]	0.50	1.25[0.65-2.41]
	**CC**	0.50	1.40[0.52-3.76]	0.27	1.83[0.62-5.35]	0.93	1.04[0.41-2.63]	0.53	1.37[0.51-3.68]	0.99	0.99[0.41-2.38]	0.30	0.66[0.30-1.46]
	**G**	-	Ref.	-	Ref.	-	Ref.	-	Ref.	-	Ref.	-	Ref.
	**C**	0.53	1.17[0.72-1.90]	0.31	1.31[0.78-2.21]	0.92	1.03[0.63-1.67]	0.57	1.14[0.73-1.78]	0.98	1.00[0.65-1.54]	0.37	0.84[0.57-1.23]
	**rs2670660**												
	**AA**	-	Ref.	-	Ref.	-	Ref.	-	Ref.	-	Ref.	-	Ref.
	**AG**	0.50	0.75[0.33-1.72]	0.72	0.85[0.36-2.03]	0.70	0.85[0.37-1.94]	0.49	0.76[0.36-1.64]	0.46	0.76[0.36-1.59]	0.96	0.98[0.50-1.91]
	**GG**	0.89	1.07[0.40-2.91]	0.74	1.20[0.40-3.59]	0.33	0.61[0.23-1.63]	0.85	1.10[0.42-2.86]	0.43	0.70[0.29-1.68]	0.29	0.64[0.28-1.45]
	**A**	-	Ref.	-	Ref.	-	Ref.	-	Ref.	-	Ref.	-	Ref.
	**G**	0.96	1.01[0.62-1.65]	0.81	1.07[0.64-1.78]	0.33	0.79[0.48-1.28]	0.94	1.02[0.65-1.59]	0.43	0.84[0.55-1.29]	0.32	0.82[0.56-1.21]

COVID-19: coronavirus 19 disease.

Table S3 continuation. Differences in *NLRP1* genotype and allele distribution between COVID-19 mild patients and moderate patients, severe patients as well as critical patients.

Locus	SNP	**COVID-19 Patients** Mild *vs* moderate		Mild *vs* Severe		Mild *vs* Critical		Moderate *vs* Severe		Moderate *vs Critical*		Severe *vs Critical*	
		p	OR [95% CI]	*p*	OR [95% CI]	*p*	OR [95% CI]	*p*	OR [95% CI]	*p*	OR [95% CI]	*p*	OR [95% CI]
NLRP1	**rs12150220**												
	**AA**	-	Ref.	-	Ref.	-	Ref.	-	Ref.	-	Ref.	-	Ref.
	**AT**	0.89	0.94[0.42-2.11]	0.47	0.73[0.31-1.72]	0.97	0.98[0.44-2.20]	0.16	0.58[0.27-1.24]	0.58	0.82[0.40-1.67]	0.41	1.33[0.68-2.58]
	**TT**	0.61	0.76[0.27-2.17]	0.72	0.81[0.25-2.62]	0.14	0.45[0.16-1.30]	0.97	1.02[0.40-2.62]	0.74	0.87[0.37-2.04]	0.82	0.91[0.40-2.06]
	**A**	-	Ref.	-	Ref.	-	Ref.	-	Ref.	-	Ref.	-	Ref.
	**T**	0.64	0.89[0.54-1.46]	0.66	0.89[0.53-1.50]	0.19	0.72[0.44-1.18]	0.88	0.97[0.62-1.50]	0.71	0.92[0.60-1.42]	0.90	0.98[0.66-1.44]
	**rs6502867**												
	**TT**	-	Ref.	-	Ref.	-	Ref.	-	Ref.	-	Ref.	-	Ref.
	**TC**	0.26	0.65[0.31-1.38]	0.44	0.73[0.33-1.62]	0.84	1.07[0.51-2.30]	0.50	1.26[0.65-2.44]	0.58	1.20[0.63-2.31]	0.79	1.08[0.61-1.93]
	**CC**	0.78	0.84[0.24-2.93]	0.12	4.05[0.71-23.13]	0.21	2.29[0.63-8.30]	0.07	4.29[0.87-21.03]	0.68	1.30[0.38-4.49]	0.37	0.53[0.13-2.14]
	**T**	-	Ref.	-	Ref.	-	Ref.	-	Ref.	-	Ref.	-	Ref.
	**C**	0.41	0.79[0.46-1.37]	0.61	1.17[0.64-2.11]	0.31	1.33[0.76-2.33]	0.15	1.47[0.87-2.40]	0.56	1.15[0.71-1.87]	0.74	0.93[0.59-1.45]

COVID-19: coronavirus 19 disease.

Table S4. Differences in *CARD8* genotype and allele distribution between COVID-19 mild patients and moderate patients, severe patients as well as critical patients.

Locus	SNP	COVID-19 Patients											
		Mild *vs* moderate		Mild *vs* Severe		Mild *vs* Critical		Moderate *vs* Severe		Moderate *vs Critical*		Severe *vs Critical*	
		p	OR [95% CI]	*p*	OR [95% CI]	*p*	OR [95% CI]	*p*	OR [95% CI]	*p*	OR [95% CI]	*p*	OR [95% CI]
CARD8	**rs11672725**												
	**CC**	-	Ref.	-	Ref.	-	Ref.	-	Ref.	-	Ref.	-	Ref.
	**CT**	0.37	1.43[0.65-3.15]	0.50	1.32[0.59-2.98]	0.39	0.73[0.35-1.51]	0.72	1.14[0.55-2.35]	0.08	0.55[0.28-1.07]	0.06	0.56[0.30-1.04]
	**TT**	0.23	0.32[0.05-2.07]	0.16	0.21[0.02-1.85]	0.25	0.31[0.04-2.28]	0.93	1.08[0.20-5.93]	0.63	0.69[0.15-3.12]	0.50	0.60[0.13-2.70]
	**C**	-	Ref.	-	Ref.	-	Ref.	-	Ref.	-	Ref.	-	Ref.
	**T**	0.90	0.96[0.51-1.79]	0.90	0.96[0.48-1.90]	0.23	0.69[0.38-1.26]	0.75	1.10[0.61-1.99]	0.13	0.66[0.38-1.13]	0.09	0.654[0.39-1.06]
	**rs6509365**												
	**AA**	-	Ref.	-	Ref.	-	Ref.	-	Ref.	-	Ref.	-	Ref.
	**AG**	0.01*	0.36[0.16-0.79]	0.008	0.34[0.15-0.76]	0.56	0.79[0.36-1.73]	0.26	0.68[0.35-1.33]	0.23	1.49[0.77-2.87]	0.01*	2.16[1.19-3.90]
	**GG**	0.63	0.76[0.24-2.38]	0.67	1.30[0.38-4.46]	0.93	0.95[0.31-2.92]	0.84	0.89[0.29-2.76]	0.76	0.85[0.30-2.42]	0.63	0.78[0.27-2.20]
	**A**	-	Ref.	-	Ref.	-	Ref.	-	Ref.	-	Ref.	-	Ref.
	**G**	0.09	0.63[0.37-1.08]	0.30	0.74[0.43-1.30]	0.70	0.90[0.52-1.56]	0.51	0.86[0.54-1.36]	0.72	1.09[0.68-1.73]	0.32	1.23[0.82-1.86]
	**rs2043211**												
	**AA**	-	Ref.	-	Ref.	-	Ref.	-	Ref.	-	Ref.	-	Ref.
	**AT**	0.02*	0.40[0.18-0.89]	0.01*	0.36[0.16-0.81]	0.56	0.79[0.37-1.72]	0.15	0.61[0.31-1.20]	0.52	1.24[0.65-2.37]	0.02*	2.01[1.12-3.63]
	**TT**	0.93	0.95[0.29-3.07]	0.66	1.32[0.38-4.54]	0.94	1.04[0.34-3.21]	0.53	0.69[0.22-2.18]	0.53	0.70[0.24-2.10]	0.72	0.83[0.29-2.37]
	**A**	-	Ref.	-	Ref.	-	Ref.	-	Ref.	-	Ref.	-	Ref.
	**T**	0.21	0.70[0.41-1.21]	0.36	0.77[0.44-1.35]	0.81	0.94[0.54-1.62]	0.25	0.76[0.47-1.22]	0.90	0.97[0.61-1.55]	0.32	1.23[0.81-1.86]

COVID-19: coronavirus 19 disease. *The statistical significance was lost after correcting for multiple testing using the Benjamini-Hochberg method for a False Discovery Rate (FDR) of 5%.

Table S5. Differences in *CASP1* genotype and allele distribution between COVID-19 mild patients and moderate patients, severe patients as well as critical patients.

Locus	SNP	COVID-19 Patients											
		Mild *vs* moderate		Mild *vs* Severe		Mild *vs* Critical		Moderate *vs* Severe		Moderate *vs Critical*		Severe *vs Critical*	
		p	OR [95% CI]	*p*	OR [95% CI]	*p*	OR [95% CI]	*p*	OR [95% CI]	*p*	OR [95% CI]	*p*	OR [95% CI]
CASP1	**rs501192**												
	**CC**	-	Ref.	-	Ref.	-	Ref.	-	Ref.	-	Ref.	-	Ref.
	**CT**	0.04	4.47[1.84-10.89]	0.20	1.68[0.76-3.70]	0.75	1.13[0.54-2.36]	0.12	0.54[0.25-1.17]	0.01*	0.38[0.18-0.81]	0.32	0.73[0.39-1.35]
	**TT**	0.73	0.73[0.12-4.33]	0.90	1.12[0.18-7.16]	0.64	1.56[0.25-9.88]	0.59	1.43[0.39-5.27]	0.44	1.71[0.43-6.79]	0.46	1.65[0.43-6.33]
	**C**	-	Ref.	-	Ref.	-	Ref.	-	Ref.	-	Ref.	-	Ref.
	**T**	0.05	1.95[1.01-3.74]	0.33	1.36[0.73-2.54]	0.64	1.15[0.64-2.10]	0.56	0.84[0.47-1.50]	0.25	0.72[0.41-1.26]	0.82	0.94[0.58-1.54]
	**rs488992**												
	**GG**	-	Ref.	-	Ref.	-	Ref.	-	Ref.	-	Ref.	-	Ref.
	**GA**	0.31	1.73[0.60-4.93]	0.47	1.46[0.52-4.06]	0.49	0.72[0.28-1.84]	0.71	1.19[0.47-3.00]	0.33	0.66[0.29-1.52]	0.24	0.64[0.30-1.36]
	**AA**	0.76	0.68[0.05-8.42]	0.66	1.87[0.11-31.39]	0.85	1.29[0.10-17.48]	0.57	1.88[0.21-16.72]	0.73	1.44[0.18-11.52]	0.94	1.08[0.14-8.16]
	**G**	-	Ref.	-	Ref.	-	Ref.	-	Ref.	-	Ref.	-	Ref.
	**A**	0.55	1.30[0.54-3.12]	0.40	1.46[0.60-3.55]	0.64	0.82[0.36-1.86]	0.50	1.31[0.60-2.85]	0.56	0.81[0.40-1.64]	0.35	0.73[0.38-1.41]

COVID-19: coronavirus 19 disease. *The statistical significance was lost after correcting for multiple testing using the Benjamini-Hochberg method for a False Discovery Rate (FDR) of 5%.

Table S6. Differences in *IL1B* genotype and allele distribution between COVID-19 mild patients and moderate patients, severe patients as well as critical patients.

Locus	SNP	COVID-19 Patients											
		**Mild *vs* moderate**		**Mild *vs* Severe**		**Mild *vs* Critical**		**Moderate *vs* Severe**		**Moderate *vs* *Critical***		**Severe *vs* *Critical***	
		p	**OR [95% CI]**	*p*	**OR [95% CI]**	*p*	**OR [95% CI]**	*p*	**OR [95% CI]**	*p*	**OR [95% CI]**	*p*	**OR [95% CI]**
	**rs1143634**												
	**GG**	-	Ref.	-	Ref.	-	Ref.	-	Ref.	-	Ref.	-	Ref.
	**GA**	0.93	1.04[0.49-2.17]	0.22	0.61[0.27-1.36]	0.19	0.60[0.28-1.29]	0.79	0.91[0.47-1.78]	0.65	0.86[0.45-1.64]	0.71	0.90[0.50-1.59]
	**AA**	0.33	2.76[0.36-21.11]	0.84	0.84[0.15-4.76]	0.79	1.27[0.22-7.28]	0.35	0.42[0.07-2.57]	0.86	0.83[0.11-6.06]	0.51	1.60[0.40-6.44]
	**G**	-	Ref.	-	Ref.	-	Ref.	-	Ref.	-	Ref.	-	Ref.
IL1B	**A**	0.56	1.20[0.65-2.20]	0.32	0.72[0.38-1.37]	0.41	0.78[0.42-1.43]	0.47	0.82[0.48-1.41]	0.66	0.89[0.52-1.52]	0.92	1.02[0.64-1.63]
	**rs16944**												
	**GG**	-	Ref.	-	Ref.	-	Ref.	-	Ref.	-	Ref.	-	Ref.
	**GA**	0.92	0.96[0.45-2.03]	0.95	1.02[0.48-2.19]	0.74	0.88[0.43-1.84]	0.72	0.88[0.45-1.73]	0.57	0.82[0.42-1.60]	0.70	0.89[0.50-1.60]
	**AA**	0.09	0.35[0.10-1.16]	0.59	0.70[0.19-2.60]	0.83	0.88[0.27-2.86]	0.28	1.76[0.63-4.89]	0.55	1.33[0.52-3.40]	0.42	0.68[0.26-1.74]
	**G**	-	Ref.	-	Ref.	-	Ref.	-	Ref.	-	Ref.	-	Ref.
	**A**	0.16	0.68[0.40-1.16]	0.74	0.91[0.52-1.58]	0.76	0.92[0.55-1.55]	0.49	1.18[0.74-1.89]	0.80	1.06[0.67-1.67]	0.43	0.85[0.56-1.28]

COVID-19: coronavirus 19 disease.

Table S7. Differences in *IL18* genotype and allele distribution between COVID-19 mild patients and moderate patients, severe patients as well as critical patients.

Locus	SNP	COVID-19 Patients											
		**Mild *vs* moderate**		**Mild *vs* Severe**		**Mild *vs* Critical**		**Moderate *vs* Severe**		**Moderate *vs* *Critical***		**Severe *vs* *Critical***	
		p	**OR [95% CI]**	*p*	**OR [95% CI]**	*p*	**OR [95% CI]**	*p*	**OR [95% CI]**	*p*	**OR [95% CI]**	*p*	**OR [95% CI]**
IL18	**rs187238**												
	**CC**	-	Ref.	-	Ref.	-	Ref.	-	Ref.	-	Ref.	-	Ref.
	**CG**	0.58	1.24[0.59-2.60]	0.03*	2.43[1.09-5.43]	0.16	1.71[0.81-3.61]	0.08	1.87[0.94-3.74]	0.41	1.32[0.68-2.54]	0.12	0.61[0.33-1.14]
	**GG**	0.98	0.99[0.27-3.55]	0.46	1.64[0.44-6.13]	0.65	1.39[0.34-5.62]	0.66	1.27[0.43-3.72]	0.44	1.58[0.50-5.03]	0.54	1.40[0.47-4.11]
	**C**	-	Ref.	-	Ref.	-	Ref.	-	Ref.	-	Ref.	-	Ref.
	**G**	0.77	1.09[0.63-1.86]	0.07	1.71[0.95-3.07]	0.24	1.39[0.80-2.42]	0.19	1.40[0.85-2.32]	0.30	1.29[0.79-2.10]	0.61	0.89[0.56-1.41]

COVID-19: coronavirus 19 disease. *The statistical significance was lost after correcting for multiple testing using the Benjamini-Hochberg method for a False Discovery Rate (FDR) of 5%.

Table S8. Differences in *NFKB1* genotype and allele distribution between COVID-19 mild patients and moderate patients, severe patients as well as critical patients.

		COVID-19 Patients											
Locus	SNP	Mild *vs* moderate		Mild *vs* Severe		Mild *vs* Critical		Moderate *vs* Severe		Moderate *vs Critical*		Severe *vs Critical*	
		p	OR [95% CI]	*p*	OR [95% CI]	*p*	OR [95% CI]	*p*	OR [95% CI]	*p*	OR [95% CI]	*p*	OR [95% CI]
	**rs28362491**												
	**ATTGATTG/ ATTGATTG**	-	Ref.	-	Ref.	-	Ref.	-	Ref.	-	Ref.	-	Ref.
NFKB1	**ATTGATTG/ ATTG**	0.78	1.11[0.53-2.36]	0.53	1.29[0.58-2.86]	0.18	1.68[0.79-3.58]	0.59	1.20[0.62-2.35]	0.11	1.70[0.89-3.26]	0.09	1.68[0.93-3.06]
	**ATTG/ ATTG**	0.02*	5.16[1.25-21.37]	0.32	1.84[0.55-6.18]	0.14	2.38[0.75-7.49]	0.18	0.45[0.14-1.44]	0.34	0.54[0.16-1.88]	0.48	1.37[0.57-3.27]
	**ATTGATTG**	-	Ref.	-	Ref.	-	Ref.	-	Ref.	-	Ref.	-	Ref.
	**ATTG**	0.12	1.50[0.89-2.51]	0.33	1.31[0.76-2.23]	0.09	1.56[0.93-2.62]	0.49	0.85[0.54-1.34]	0.83	1.05[0.66-1.66]	0.20	1.31[0.87-1.97]

COVID-19: coronavirus 19 disease. *The statistical significance was lost after correcting for multiple testing using the Benjamini-Hochberg method for a False Discovery Rate (FDR) of 5%.

Table S9. Differences in *ATG16L1* genotype and allele distribution between COVID-19 mild patients and moderate patients, severe patients as well as critical patients.

Locus	SNP	COVID-19 Patients											
		Mild *vs* moderate		Mild *vs* Severe		Mild *vs* Critical		Moderate *vs* Severe		Moderate *vs* *Critical*		Severe *vs* *Critical*	
		p	OR [95% CI]	*p*	OR [95% CI]	*p*	OR [95% CI]	*p*	OR [95% CI]	*p*	OR [95% CI]	*p*	OR [95% CI]
ATG16L1	**rs2241880**												
	**GG**	-	Ref.	-	Ref.	-	Ref.	-	Ref.	-	Ref.	-	Ref.
	**GA**	0.69	1.20[0.50-2.91]	0.95	1.03[0.41-2.55]	0.75	1.15[0.48-2.74]	0.92	0.96[0.45-2.05]	0.83	1.09[0.52-2.28]	0.80	1.09[0.57-2.07]
	**AA**	0.98	0.99[0.37-2.62]	0.28	1.79[0.63-5.06]	0.23	1.83[0.68-4.96]	0.53	1.32[0.55-3.17]	0.29	1.59[0.68-3.73]	0.98	0.99[0.45-2.15]
	**G**	-	Ref.	-	Ref.	-	Ref.	-	Ref.	-	Ref.	-	Ref.
	**A**	0.95	0.98[0.61-1.60]	0.28	1.33[0.80-2.21]	0.23	1.34[0.83-2.19]	0.54	1.15[0.74-1.78]	0.29	1.26[0.82-1.94]	1.00	1.00[0.68-1.47]
	**rs6754677**												
	**AA**	-	Ref.	-	Ref.	-	Ref.	-	Ref.	-	Ref.	-	Ref.
	**AG**	0.75	1.13[0.52-2.45]	0.26	1.66[0.73-3.79]	0.81	1.11[0.50-2.45]	0.28	1.45[0.74-2.84]	0.68	1.15[0.59-2.23]	0.39	0.77[0.43-1.38]
	**GG**	0.55	1.37[0.49-3.81]	0.01*	4.31[1.36-13.62]	0.01*	4.03[1.31-12.36]	0.44	1.48[0.54-4.01]	0.46	1.44[0.55-3.77]	0.42	0.68[0.27-1.73]
	**A**	-	Ref.	-	Ref.	-	Ref.	-	Ref.	-	Ref.	-	Ref.
	**G**	0.55	1.16[0.71-1.91]	0.01*	1.96[1.15-3.34]	0.04*	1.68[1.02-2.79]	0.30	1.28[0.81-2.02]	0.47	1.18[0.76-1.83]	0.32	0.81[0.54-1.22]

COVID-19: coronavirus 19 disease. *The statistical significance was lost after correcting for multiple testing using the Benjamini-Hochberg method for a False Discovery Rate (FDR) of 5%.

Table S10. Differences in *MIF* genotype and allele distribution between COVID-19 mild patients and moderate patients, severe patients as well as critical patients.

Locus	SNP	**COVID-19 Patients**											
		**Mild *vs* moderate**		**Mild *vs* Severe**		**Mild *vs* Critical**		**Moderate *vs* Severe**		**Moderate *vs* *Critical***		**Severe *vs* *Critical***	
		p	**OR [95% CI]**	*p*	**OR [95% CI]**	*p*	**OR [95% CI]**	*p*	**OR [95% CI]**	*p*	**OR [95% CI]**	*p*	**OR [95% CI]**
	**rs755622**												
	**GG**	-	Ref.	-	Ref.	-	Ref.	-	Ref.	-	Ref.	-	Ref.
MIF	**GC**	0.19	1.70[0.77-3.72]	0.90	1.05[0.49-2.29]	0.44	1.35[0.63-2.91]	0.22	0.63[0.30-1.32]	0.48	0.77[0.37-1.59]	0.44	1.27[0.69-2.37]
	**CC**	0.56	0.50[0.05-5.20]	0.73	1.77[0.07-45.80]	0.52	0.52[0.07-3.84]	0.29	4.02[0.32-51.01]	0.79	0.77[0.12-4.83]	0.22	0.24[0.03-2.30]
	**G**	-	Ref.	-	Ref.	-	Ref.	-	Ref.	-	Ref.	-	Ref.
	**C**	0.43	1.31[0.67-2.56]	0.82	1.08[0.55-2.10]	0.85	1.07[0.56-2.02]	0.60	0.85[0.45-1.58]	0.48	0.80[0.44-1.47]	0.95	0.98[0.58-1.67]

COVID-19: coronavirus 19 disease.

Table S11. Differences in *NLRP3* and *NLRC4* genotype and allele distribution between COVID-19 patients and healthy controls.

Locus	SNP	Mild COVID-19 patients *vs* Healthy Controls		Moderate COVID-19 patients *vs* Healthy Controls		Severe COVID-19 patients *vs* Healthy Controls		Critical COVID-19 patients *vs* Healthy Controls	
		p	OR [95% CI]	*p*	OR [95% CI]	*p*	OR [95% CI]	*p*	OR [95% CI]
NLRP3	**rs4925659**								
	**GG**	-	Ref.	-	Ref.	-	Ref.	-	Ref.
	**GA**	0.12	1.66 [0.87-3.14]	0.18	0.68 [0.39-1.19]	0.79	0.93 [0.55-1.58]	0.57	0.87 [0.53-1.42]
	**AA**	0.02*	2.64 [1.15-6.07]	0.84	1.09 [0.50-2.38]	0.45	1.34 [0.63-2.83]	0.53	1.26 [0.62-2.55]
	**G**	-	Ref.	-	Ref.	-	Ref.	-	Ref.
	**A**	0.03*	1.56 [1.05-2.30]	0.71	0.93 [0.64-1.36]	0.62	1.09 [0.77-1.56]	0.77	1.05 [0.75-1.47]
	**rs10159239**								
	**AA**	-	Ref.	-	Ref.	-	Ref.	-	Ref.
	**AG**	0.42	0.78 [0.43-1.43]	0.15	0.63 [0.34-1.18]	0.40	1.29 [0.71-2.36]	0.19	1.47 [0.83-2.59]
	**GG**	0.05	0.43 [0.18-1.01]	0.40	1.33 [0.68-2.61]	0.56	1.24 [0.60-2.54]	0.40	1.34 [0.68-2.64]
	**A**	-	Ref.	-	Ref.	-	Ref.	-	Ref.
	**G**	0.07	0.69 [0.47-1.03]	0.41	1.17 [0.81-1.68]	0.55	1.11 [0.79-1.57]	0.40	1.15 [0.83-1.59]
	**rs10754558**								
	**CC**	-	Ref.	-	Ref.	-	Ref.	-	Ref.
	**CG**	0.06	0.57 [0.31-1.03]	0.14	0.63 [0.34-1.17]	0.30	1.35 [0.76-2.40]	0.28	1.35 [0.78-2.32]
	**GG**	0.03*	0.38 [0.16-0.92]	0.18	1.58 [0.81-3.09]	0.69	1.16 [0.56-2.39]	0.65	1.17 [0.59-2.31]
	**C**	-	Ref.	-	Ref.	-	Ref.	-	Ref.
	**G**	0.02*	0.61 [0.41-0.92]	0.21	1.26 [0.88-1.82]	0.61	1.09 [0.78-1.54]	0.58	1.10 [0.79-1.52]
	**rs4353135**								
	**TT**	-	Ref.	-	Ref.	-	Ref.	-	Ref.
	**TG**	0.08	0.58 [0.32-1.06]	0.41	1.28 [0.71-2.30]	0.41	1.26 [0.72-2.20]	0.29	0.76 [0.45-1.27]
	**GG**	0.48	0.74 [0.32-1.72]	0.69	1.19 [0.52-2.71]	0.67	1.19 [0.54-2.59]	0.45	1.29 [0.66-2.53]
	**T**	-	Ref.	-	Ref.	-	Ref.	-	Ref.
	**G**	0.19	0.76 [0.51-1.15]	0.58	1.11 [0.77-1.61]	0.56	1.11 [0.78-1.58]	0.73	1.06 [0.76-1.48]
NLRC4	**rs385076**								
	**CC**	-	Ref.	-	Ref.	-	Ref.	-	Ref.
	**CT**	0.58	0.85 [0.47-1.52]	0.31	0.74 [0.42-1.31]	0.83	1.06 [0.62-1.81]	0.06	0.62 [0.38-1.02]
	**TT**	0.40	0.68 [0.27-1.67]	0.66	1.19 [0.56-2.51]	0.99	1.01 [0.47-2.17]	0.08	0.51 [0.24-1.10]
	**C**	-	Ref.	-	Ref.	-	Ref.	-	Ref.
	**T**	0.35	0.83 [0.55-1.23]	0.95	1.01 [0.70-1.47]	0.94	1.01 [0.72-1.44]	0.04*	0.70 [0.50-0.98]
	**rs479333**								
	**GG**	-	Ref.	-	Ref.	-	Ref.	-	Ref.
	**GC**	0.67	0.88 [0.48-1.60]	0.20	0.68 [0.37-1.22]	0.82	1.07 [0.61-1.86]	0.04*	0.59 [0.36-0.98]
	**CC**	0.56	0.77 [0.33-1.81]	0.52	1.27 [0.62-2.61]	1.00	1.00 [0.47-2.14]	0.22	0.65 [0.32-1.30]
	**G**	-	Ref.	-	Ref.	-	Ref.	-	Ref.
	**C**	0.50	0.87 [0.59-1.29]	0.77	1.06 [0.73-1.53]	0.95	1.01 [0.71-1.43]	0.10	0.75 [0.54-1.05]

COVID-19: coronavirus 19 disease. *The statistical significance was lost after correcting for multiple testing using the Benjamini-Hochberg method for a False Discovery Rate (FDR) of 5%.

Table S12. Differences in *NLRP1* genotype and allele distribution between COVID-19 patients and healthy controls.

Locus	SNP	Mild COVID-19 patients *vs* Healthy Controls		Moderate COVID-19 patients *vs* Healthy Controls		Severe COVID-19 patients *vs* Healthy Controls		Critical COVID-19 patients *vs* Healthy Controls	
		p	OR [95% CI]	*p*	OR [95% CI]	*p*	OR [95% CI]	*p*	OR [95% CI]
NLRP1	**rs4790797**								
	**GG**	-	Ref.	-	Ref.	-	Ref.	-	Ref.
	**GA**	0.68	1.15 [0.60-2.21]	0.14	1.63 [0.86-3.10]	0.38	1.30 [0.72-2.31]	0.97	0.99 [0.57-1.72]
	**AA**	0.69	1.17 [0.55-2.50]	0.70	1.16 [0.54-2.53]	0.47	0.76 [0.36-1.59]	0.64	1.16 [0.62-2.17]
	**G**	-	Ref.	-	Ref.	-	Ref.	-	Ref.
	**A**	0.63	1.10 [0.75-1.62]	0.65	1.09 [0.75-1.58]	0.58	0.91 [0.64-1.29]	0.64	1.08 [0.78-1.50]
	**rs8182352**								
	**TT**	-	Ref.	-	Ref.	-	Ref.	-	Ref.
	**TC**	0.72	1.13 [0.59-2.15]	0.08	1.77 [0.93-3.34]	0.33	1.33 [0.75-2.35]	0.80	1.07 [0.62-1.85]
	**CC**	0.86	1.07 [0.51-2.25]	0.77	1.12 [0.52-2.41]	0.43	0.75 [0.36-1.53]	0.80	1.08 [0.58-2.01]
	**T**	-	Ref.	-	Ref.	-	Ref.	-	Ref.
	**C**	0.79	1.05 [0.72-1.55]	0.71	1.07 [0.75-1.54]	0.53	0.90 [0.63-1.27]	0.79	1.05 [0.76-1.44]
	**rs878329**								
	**GG**	-	Ref.	-	Ref.	-	Ref.	-	Ref.
	**GC**	0.74	1.12 [0.58-2.15]	0.08	1.77 [0.94-3.31]	0.18	1.48 [0.83-2.63]	0.76	1.09 [0.63-1.88]
	**CC**	0.75	1.12 [0.54-2.34]	0.88	1.06 [0.50-2.26]	0.40	0.73 [0.35-1.51]	0.73	1.11 [0.60-2.06]
	**G**	-	Ref.	-	Ref.	-	Ref.	-	Ref.
	**C**	0.68	1.08 [0.74-1.60]	0.80	1.05 [0.73-1.51]	0.53	0.89 [0.63-1.27]	0.71	1.06 [0.77-1.47]
	**rs2670660**								
	**AA**	-	Ref.	-	Ref.	-	Ref.	-	Ref.
	**AG**	0.76	1.10 [0.59-2.07]	0.25	1.43 [0.78-2.63]	0.13	1.57 [0.88-2.80]	0.19	1.45 [0.83-2.53]
	**GG**	0.68	0.85 [0.40-1.82]	0.95	1.02 [0.49-2.12]	0.66	0.85 [0.41-1.75]	0.43	1.29 [0.68-2.44]
	**A**	-	Ref.	-	Ref.	-	Ref.	-	Ref.
	**G**	0.76	0.94 [0.64-1.39]	0.87	1.03 [0.72-1.48]	0.82	0.96 [0.68-1.36]	0.39	1.15 [0.83-1.59]
	**rs12150220**								
	**AA**	-	Ref.	-	Ref.	-	Ref.	-	Ref.
	**AT**	0.69	1.14 [0.61-2.10]	0.63	1.16 [0.64-2.10]	0.09	1.66 [0.92-2.99]	0.50	1.20 [0.70-2.06]
	**TT**	0.20	0.58 [0.25-1.34]	0.91	0.96 [0.47-1.97]	0.97	0.99 [0.47-2.06]	0.77	1.10 [0.58-2.07]
	**A**	-	Ref.	-	Ref.	-	Ref.	-	Ref.
	**T**	0.31	0.81 [0.55-1.21]	0.96	0.99 [0.69-1.43]	0.87	1.03 [0.73-1.46]	0.73	1.06 [0.77-1.47]
	**rs6502867**								
	**TT**	-	Ref.	-	Ref.	-	Ref.	-	Ref.
	**TC**	0.73	1.11 [0.61-2.02]	0.08	1.62 [0.94-2.81]	0.10	1.55 [0.92-2.59]	0.19	1.39 [0.85-2.27]
	**CC**	0.14	2.19 [0.77-6.29]	0.16	2.14 [0.75-6.12]	0.67	0.75 [0.19-2.88]	0.36	1.60 [0.59-4.37]
	**T**	-	Ref.	-	Ref.	-	Ref.	-	Ref.
	**C**	0.27	1.29 [0.82-2.03]	0.04*	1.53 [1.01-2.32]	0.35	1.22 [0.81-1.84]	0.15	1.33 [0.90-1.94]

COVID-19: coronavirus 19 disease. *The statistical significance was lost after correcting for multiple testing using the Benjamini-Hochberg method for a False Discovery Rate (FDR) of 5%.

Table S13. Differences in *CARD8*, and *CASP1* genotype and allele distribution between COVID-19 patients and healthy controls.

Locus	SNP	Mild COVID-19 patients *vs* Healthy Controls		Moderate COVID-19 patients *vs* Healthy Controls		Severe COVID-19 patients *vs* Healthy Controls		Critical COVID-19 patients *vs* Healthy Controls	
		p	OR [95% CI]	*p*	OR [95% CI]	*p*	OR [95% CI]	*p*	OR [95% CI]
CARD8	**rs11672725**								
	**CC**	-	Ref.	-	Ref.	-	Ref.	-	Ref.
	**CT**	0.44	1.26[0.70-2.28]	0.81	0.93[0.52-1.67]	0.63	0.87[0.50-1.52]	0.07	1.57[0.96-2.57]
	**TT**	0.57	0.63[0.13-3.04]	0.68	1.27[0.41-3.96]	0.50	0.63[0.16-2.39]	0.94	1.05[0.34-3.24]
	**C**	-	Ref.	-	Ref.	-	Ref.	-	Ref.
	**T**	0.79	1.07[0.66-1.74]	0.91	1.03[0.65-1.63]	0.43	0.83[0.52-1.32]	0.18	1.31[0.88-1.94]
	**rs6509365**								
	**AA**	-	Ref.	-	Ref.	-	Ref.	-	Ref.
	**AG**	0.01*	0.43[0.24-0.79]	0.50	0.83[0.48-1.42]	0.28	1.33[0.79-2.23]	0.06	0.62[0.38-1.02]
	**GG**	0.60	0.78[0.32-1.94]	0.75	0.86[0.35-2.15]	0.89	0.94[0.37-2.35]	0.77	0.89[0.41-1.94]
	**A**	-	Ref.	-	Ref.	-	Ref.	-	Ref.
	**G**	0.06	0.66[0.43-1.02]	0.58	0.90[0.61-1.32]	0.67	1.08[0.76-1.55]	0.25	0.81[0.57-1.16]
	**rs2043211**								
	**AA**	-	Ref.	-	Ref.	-	Ref.	-	Ref.
	**AT**	0.01*	0.44[0.24-0.80]	0.24	0.73[0.42-1.24]	0.28	1.33[0.80-2.23]	0.09	0.66[0.41-1.07]
	**TT**	0.74	0.86[0.74-2.13]	0.55	0.75[0.29-1.94]	1.00	1.00[0.40-2.51]	0.74	0.87[0.39-1.94]
	**A**	-	Ref.	-	Ref.	-	Ref.	-	Ref.
	**T**	0.10	0.70[0.45-1.07]	0.31	0.81[0.55-1.21]	0.59	1.11[0.77-1.58]	0.27	0.82[0.58-1.16]
CASP1	**rs501192**								
	**CC**	-	Ref.	-	Ref.	-	Ref.	-	Ref.
	**CT**	0.45	1.25 [0.70-2.22]	0.04*	0.50 [0.27-0.96]	0.52	0.84 [0.49-1.44]	0.77	1.08 [0.66-1.76]
	**TT**	0.65	1.49 [0.26-8.42]	0.09	3.13 [0.85-11.49]	0.11	2.88 [0.79-10.59]	0.46	1.71 [0.42-7.05]
	**C**	-	Ref.	-	Ref.	-	Ref.	-	Ref.
	**T**	0.40	1.23 [0.76-1.98]	0.64	0.89 [0.55-1.45]	0.65	1.11 [0.72-1.71]	0.55	1.13 [0.75-1.71]
	**rs488992**								
	**GG**	-	Ref.	-	Ref.	-	Ref.	-	Ref.
	**GA**	0.11	0.54 [0.26-1.14]	0.12	0.57 [0.28-1.15]	0.05	0.50 [0.26-0.99]	0.41	0.79 [0.45-1.39]
	**AA**	0.89	1.19 [0.11-13.35]	0.46	2.10 [0.29-15.23]	0.60	1.71 [0.24-12.33]	0.68	1.51 [0.21-10.91]
	**G**	-	Ref.	-	Ref.	-	Ref.	-	Ref.
	**A**	0.18	0.64 [0.33-1.24]	0.33	0.74 [0.41-1.35]	0.14	0.65 [0.36-1.16]	0.60	0.87 [0.53-1.44]

COVID-19: coronavirus 19 disease. *The statistical significance was lost after correcting for multiple testing using the Benjamini-Hochberg method for a False Discovery Rate (FDR) of 5%.

Table S14. Differences in *IL1B, IL18, NFKB1, ATG16L1*, and *MIF* genotype and allele distribution between COVID-19 patients and healthy controls.

Locus	SNP	Mild COVID-19 patients *vs* Healthy Controls		Moderate COVID-19 patients *vs* Healthy Controls		Severe COVID-19 patients *vs* Healthy Controls		Critical COVID-19 patients *vs* Healthy Controls	
		p	OR [95% CI]	*p*	OR [95% CI]	*p*	OR [95% CI]	*p*	OR [95% CI]
IL1B	**rs1143634**								
	**GG**	-	Ref.	-	Ref.	-	Ref.	-	Ref.
	**GA**	0.85	0.94 [0.53-1.70]	0.91	0.97 [0.56-1.68]	0.96	0.99 [0.58-1.67]	0.75	1.08 [0.66-1.76]
	**AA**	0.37	0.55 [0.15-2.04]	0.14	0.31 [0.07-1.44]	0.49	0.68 [0.23-2.00]	0.19	0.46 [0.14-1.47]
	**G**	-	Ref.	-	Ref.	-	Ref.	-	Ref.
	**A**	0.38	0.81 [0.50-1.30]	0.28	0.78 [0.50-1.22]	0.61	0.90 [0.59-1.36]	0.52	0.88 [0.60-1.30]
	**rs16944**								
	**GG**	-	Ref.	-	Ref.	-	Ref.	-	Ref.
	**GA**	0.45	0.80 [0.45-1.43]	0.35	0.77 [0.43-1.35]	0.70	0.90 [0.54-1.51]	0.71	0.91 [0.55-1.49]
	**AA**	0.59	0.75 [0.27-2.08]	0.24	1.63 [0.73-3.64]	0.75	0.87 [0.36-2.10]	0.56	1.26 [0.58-2.74]
	**G**	-	Ref.	-	Ref.	-	Ref.	-	Ref.
	**A**	0.46	0.85 [0.56-1.30]	0.60	1.11 [0.75-1.64]	0.67	0.92 [0.63-1.34]	0.80	1.05 [0.74-1.48]
IL18	**rs187238**								
	**CC**	-	Ref.	-	Ref.	-	Ref.	-	Ref.
	**CG**	0.62	1.16 [0.65-2.06]	0.93	0.97 [0.56-1.70]	0.02*	0.52 [0.30-0.90]	0.55	0.86 [0.53-1.41]
	**GG**	0.78	0.86 [0.29-2.51]	0.62	1.26 [0.51-3.07]	0.68	0.83 [0.35-1.99]	0.35	0.64 [0.25-1.63]
	**C**	-	Ref.	-	Ref.	-	Ref.	-	Ref.
	**G**	0.89	1.03 [0.67-1.59]	0.75	1.07 [0.71-1.60]	0.10	0.71 [0.47-1.06]	0.31	0.82 [0.56-1.20]
NFKB1	**rs28362491**								
	**ATTGATTG/ ATTGATTG**	-	Ref.	-	Ref.	-	Ref.	-	Ref.
	**ATTGATTG/ ATTG**	0.72	1.12 [0.62-2.01]	0.92	0.97 [0.57-1.67]	0.59	0.87 [0.51-1.46]	0.03*	0.58 [0.36-0.95]
	**ATTG/ ATTG**	0.92	1.05 [0.41-2.65]	0.17	0.48 [0.17-1.38]	0.68	1.17 [0.54-2.55]	0.63	0.83 [0.40-1.75]
	**ATTGATTG**	-	Ref.	-	Ref.	-	Ref.	-	Ref.
	**ATTG**	0.78	1.06 [0.71-1.57]	0.34	0.83 [0.57-1.22]	0.93	1.02 [0.71-1.45]	0.19	0.80 [0.57-1.12]
ATG16L1	**rs2241880**								
	**GG**	-	Ref.	-	Ref.	-	Ref.	-	Ref.
	**GA**	0.28	1.46 [0.74-2.91]	0.76	1.10 [0.59-2.07]	0.79	1.08 [0.61-1.91]	0.92	0.97 [0.57-1.66]
	**AA**	0.05	2.25 [1.01-5.01]	0.10	1.85 [0.89-3.86]	0.52	1.27 [0.62-2.59]	0.56	1.22 [0.63-2.38]
	**G**	-	Ref.	-	Ref.	-	Ref.	-	Ref.
	**A**	0.04*	1.50 [1.01-2.21]	0.11	1.35 [0.93-1.95]	0.54	1.12 [0.79-1.58]	0.62	1.09 [0.78-1.51]
	**rs6754677**								
	**AA**	-	Ref.	-	Ref.	-	Ref.	-	Ref.
	**AG**	0.46	1.26 [0.68-2.32]	0.60	1.16 [0.66-2.04]	0.49	0.83 [0.50-1.40]	0.90	0.99 [0.59-1.58]
	**GG**	0.09	2.05 [0.90-4.64]	0.36	1.46 [0.65-3.26]	0.64	0.82 [0.36-1.85]	0.88	0.94 [0.44-2.01]
	**A**	-	Ref.	-	Ref.	-	Ref.	-	Ref.
	**G**	0.09	1.41 [0.95-2.09]	0.36	1.19 [0.82-1.73]	0.51	0.88 [0.61-1.27]	0.87	0.97 [0.69-1.36]
MIF	**rs755622**								
	**GG**	-	Ref.	-	Ref.	-	Ref.	-	Ref.
	**GC**	0.24	1.44 [0.79-2.61]	0.70	0.89 [0.48-1.63]	0.45	1.23 [0.72-2.13]	0.94	0.98 [0.57-1.67]
	**CC**	0.83	0.84 [0.17-4.18]	0.57	0.63 [0.13-3.10]	0.25	0.29 [0.03-2.37]	0.88	0.91 [0.26-3.19]
	**G**	-	Ref.	-	Ref.	-	Ref.	-	Ref.
	**C**	0.53	1.18 [0.71-1.95]	0.51	0.84 [0.50-1.41]	0.94	0.98 [0.61-1.57]	0.88	0.97 [0.62-1.51]

COVID-19: coronavirus 19 disease. *The statistical significance was lost after correcting for multiple testing using the Benjamini-Hochberg method for a False Discovery Rate (FDR) of 5%.

Table S15. Differences in *NLRP3, NLRC4, NLRP1, CARD8, CASP1, IL1B,* and *ATG16L1* haplotype distribution between COVID-19 mild patients and moderate patients, severe patients as well as critical patients.

Locus	SNP	Mild *vs* moderate		Mild *vs* Severe		Mild *vs* Critical		Moderate *vs* Severe		Moderate *vs Critical*		Severe *vs Critical*	
		p	OR [95% CI]	*p*	OR [95% CI]	*p*	OR [95% CI]	*p*	OR [95% CI]	*p*	OR [95% CI]	*p*	OR [95% CI]
NLRP3[1]	**AACT**	-	Ref.	-	Ref.	-	Ref.	-	Ref.	-	Ref.	-	Ref.
	**GGGT**	0.15	0.62 [0.30-1.25]	0.14	0.62 [0.31-1.23]	0.02*	0.47 [0.24-0.91]	0.96	1.01 [0.53-1.94]	0.38	0.77 [0.42-1.43]	0.43	0.76 [0.43-1.36]
	**GGGG**	<0.01*	0.43 [0.22-0.82]	0.02*	0.49 [0.26-0.82]	0.02*	0.48 [0.26-0.91]	0.64	1.14 [0.64-2.02]	0.64	1.13 [0.65-1.98]	0.98	0.99 [0.58-1.71]
	**AACG**	0.75	1.16 [0.43-3.24]	0.45	0.73 [0.30-1.74]	0.11	0.54 [0.23-1.21]	0.29	0.63 [0.24-1.60]	0.06	0.47 [0.18-1.11]	0.38	0.74 [0.35-1.53]
	**GACG**	0.95	0.97 [0.36-2.67]	0.88	1.07 [0.40-2.85]	0.87	1.07 [0.41-2.79]	0.83	1.10 [0.40-3.00]	0.82	1.11 [0.41-2.93]	1.00	1.00 [0.38-2.59]
	**GACT**	0.29	0.59 [0.19-1.74]	0.76	0.86 [0.27-2.62]	0.31	0.62 [0.21-1.71]	0.43	1.44 [0.52-4.07]	0.92	1.04 [0.41-2.64]	0.47	0.72 [0.27-1.88]
NLRC4[2]	**CG**	-	Ref.	-	Ref.	-	Ref.	-	Ref.	-	Ref.	-	Ref.
	**TC**	0.41	0.82 [0.50-1.35]	0.50	0.86 [0.53-1.38]	0.39	1.22 [0.76-1.94]	0.85	1.04 [0.67-1.63]	0.06	1.48 [0.95-2.29]	0.08	1.42 [0.94-2.15]
NLRP1[3]	**TTGCCA**	-	Ref.	-	Ref.	-	Ref.	-	Ref.	-	Ref.	-	Ref.
	**TAAGTG**	0.71	0.90 [0.49-1.65]	0.90	1.04 [0.59-1.83]	0.54	0.85 [0.49-1.48]	0.59	1.15 [0.67-1.99]	0.82	0.94 [0.56-1.61]	0.40	0.82 [0.50-1.34]
	**CAAGTG**	0.90	1.04 [0.53-2.07]	1.00	1.00 [0.52-1.95]	0.67	0.88 [0.46-1.68]	0.90	0.96 [0.52-1.79]	0.55	0.84 [0.47-1.54]	0.63	0.88 [0.50-1.55]
CARD8[4]	**CAA**	-	Ref.	-	Ref.	-	Ref.	-	Ref.	-	Ref.	-	Ref.
	**CGT**	0.28	0.75 [0.42-1.32]	0.02*	0.55 [0.32-0.95]	0.25	0.75 [0.43-1.27]	0.21	0.74 [0.45-1.22]	1.00	1.00 [0.61-1.64]	0.17	1.35 [0.86-2.13]
	**TAA**	0.59	0.84 [0.43-1.65]	0.58	0.84 [0.43-1.62]	0.15	0.66 [0.36-1.21]	1.00	1.00 [0.54-1.86]	0.38	0.79 [0.44-1.38]	0.36	0.79 [0.45-1.36]
CASP1[5]	**CG**	-	Ref.	-	Ref.	-	Ref.	-	Ref.	-	Ref.	-	Ref.
	**TA**	0.87	0.94 [0.39-2.20]	0.99	1.01 [0.42-2.33]	0.46	0.76 [0.34-1.63]	0.84	1.07 [0.49-2.36]	0.54	0.82 [0.39-1.64]	0.40	0.76 [0.37-1.50]
	**TG**	0.08	1.98 [0.87-4.65]	0.62	1.18 [0.58-2.38]	0.19	1.55 [0.75-3.17]	0.16	0.60 [0.26-1.30]	0.52	0.79 [0.34-1.74]	0.39	1.32 [0.67-2.61]
IL1B[6]	**GG**	-	Ref.	-	Ref.	-	Ref.	-	Ref.	-	Ref.	-	Ref.
	**AG**	0.46	0.82 [0.48-1.42]	0.96	1.01 [0.59-1.72]	0.71	0.91 [0.55-1.52]	0.39	1.23 [0.75-2.03]	0.65	1.11 [0.69-1.79]	0.65	0.90 [0.57-1.43]
	**GA**	0.76	1.10 [0.58-2.09]	0.85	1.06 [0.58-1.93]	0.74	1.10 [0.61-1.97]	0.90	0.96 [0.53-1.75]	1.00	1.00 [0.56-1.79]	0.88	1.04 [0.60-1.78]
ATG16L1[7]	**GA**	-	Ref.	-	Ref.	-	Ref.	-	Ref.	-	Ref.	-	Ref.
	**AG**	0.54	1.16 [0.69-1.95]	0.07	1.55 [0.94-2.57]	0.12	1.43 [0.89-2.30]	0.22	1.33 [0.82-2.18]	0.35	1.23 [0.78-1.95]	0.71	0.92 [0.59-1.44]
	**AA**	0.77	0.90 [0.42-1.91]	0.65	0.86 [0.42-1.73]	0.44	1.30 [0.62-2.63]	0.88	0.95 [0.50-1.82]	0.25	1.44 [0.74-2.77]	0.16	1.50 [0.82-2.77]

Column group heading: **COVID-19 Patients**

COVID-19: coronavirus 19 disease. The order of the polymorphisms was: [1]rs4925659, rs10159239, rs10754558 and rs4353135 for *NLRP3*; [2]rs385076 and rs479333 for *NLRC4*; [3]rs4790797, rs8182352, rs878329, rs2670660, rs12150220, rs6502867 for *NLRP1*; [4]rs11672725, rs6509365 and rs2043211 for *CARD8*; [5]rs501192 and rs488992 for *CASP1*; [6]rs1143634 and rs16944 for *IL1B*; [7]rs2241880 and rs6754677 for *ATG16L1*. Haplotypes with a frequency higher than 5% are displayed in the table. *The statistical significance was lost after correcting for multiple testing using the Benjamini-Hochberg method for a False Discovery Rate (FDR) of 5%.

Table S16. Differences in *NLRP3, NLRC4, NLRP1, CARD8, CASP1, IL1B,* and *ATG16L1* haplotype distribution between COVID-19 patients and healthy controls.

Locus	Haplotypes	Mild COVID-19 patients *vs* Healthy Controls		Moderate COVID-19 patients *vs* Healthy Controls		Severe COVID-19 patients *vs* Healthy Controls		Critical COVID-19 patients *vs* Healthy Controls	
		p	**OR [95% CI]**	***p***	**OR [95% CI]**	***p***	**OR [95% CI]**	***p***	**OR [95% CI]**
NLRP3[1]	**AACT**	-	Ref.	-	Ref.	-	Ref.	-	Ref.
	**GGGT**	<0.01*	0.42 [0.23-0.77]	0.16	0.69 [0.39-1.20]	0.13	0.68 [0.40-1.15]	0.62	0.89 [0.55-1.45]
	**GGGG**	0.04*	0.56 [0.31-1.02]	0.27	1.32 [0.78-2.21]	0.55	1.16 [0.70-1.91]	0.52	1.16 [0.71-1.89]
	**AACG**	0.08	0.54 [0.24-1.13]	0.05	0.47 [0.19-1.04]	0.33	0.73 [0.37-1.43]	0.99	1.00 [0.54-1.83]
	**GACG**	0.28	0.67 [0.29-1.47]	0.34	0.69 [0.29-1.55]	0.21	0.62 [0.27-1.36]	0.19	0.62 [0.28-1.33]
	**GACT**	0.12	0.51 [0.19-1.25]	0.69	0.86 [0.36-1.92]	0.19	0.59 [0.24-1.37]	0.58	0.82 [0.38-1.74]
NLRC4[2]	**CG**	-	Ref.	-	Ref.	-	Ref.	-	Ref.
	**TC**	0.50	0.86 [0.55-1.36]	0.81	1.05 [0.69-1.61]	0.96	1.01 [0.67-1.51]	0.08	0.71 [0.48-1.06]
NLRP1[3]	**TTGCCA**	-	Ref.	-	Ref.	-	Ref.	-	Ref.
	**TAAGTG**	0.95	1.02 [0.61-1.68]	0.70	0.91 [0.56-1.47]	0.81	1.05 [0.68-1.63]	0.47	0.86 [0.57-1.31]
	**CAAGTG**	0.52	1.20 [0.63-2.16]	0.40	1.25 [0.72-2.15]	0.46	1.20 [0.71-2.01]	0.83	1.05 [0.64-1.72]
CARD8[4]	**CAA**	-	Ref.	-	Ref.	-	Ref.	-	Ref.
	**CGT**	0.05	0.63 [0.38-1.03]	0.44	0.85 [0.54-1.32]	0.50	1.14 [0.76-1.70]	0.39	0.85 [0.57-1.26]
	**TAA**	0.56	0.85 [0.47-1.51]	0.96	1.01 [0.58-1.73]	0.97	1.01 [0.60-1.69]	0.26	1.29 [0.81-2.03]
CASP1[5]	**CG**	-	Ref.	-	Ref.	-	Ref.	-	Ref.
	**TA**	0.28	0.69 [0.32-1.38]	0.32	0.74 [0.38-1.38]	0.21	0.69 [0.36-1.27]	0.71	0.91 [0.53-1.54]
	**TG**	0.01*	2.29 [1.13-4.57]	0.69	1.16 [0.51-2.51]	0.03*	1.94 [1.01-3.74]	0.22	1.47 [0.75-2.86]
IL1B[6]	**GG**	-	Ref.	-	Ref.	-	Ref.	-	Ref.
	**AG**	0.96	0.99 [0.61-1.59]	0.39	1.20 [0.77-1.86]	0.90	0.97 [0.63-1.49]	0.69	1.08 [0.73-1.60]
	**GA**	0.90	1.03 [0.60-1.76]	0.81	0.94 [0.55-1.60]	0.92	0.98 [0.60-1.59]	0.79	0.94 [0.59-1.50]
ATG16L1[7]	**GA**	-	Ref.	-	Ref.	-	Ref.	-	Ref.
	**AG**	0.06	1.50 [0.96-2.33]	0.22	1.29 [0.84-1.97]	0.86	0.97 [0.64-1.45]	0.81	1.05 [0.72-1.52]
	**AA**	0.57	1.19 [0.61-2.27]	0.33	1.32 [0.72-2.39]	0.21	1.39 [0.80-2.37]	0.75	0.92 [0.53-1.59]

COVID-19: coronavirus 19 disease. The order of the polymorphisms was: [1]rs4925659, rs10159239, rs10754558 and rs4353135 for *NLRP3*; [2]rs385076 and rs479333 for *NLRC4*; [3]rs4790797, rs8182352, rs878329, rs2670660, rs12150220, rs6502867 for *NLRP1*; [4]rs11672725, rs6509365 and rs2043211 for *CARD8*; [5]rs501192 and rs488992 for *CASP1*; [6]rs1143634 and rs16944 for *IL1B*; [7]rs2241880 and rs6754677 for *ATG16L1*. Haplotypes with a frequency higher than 5% are displayed in the table. *The statistical significance was lost after correcting for multiple testing using the Benjamini-Hochberg method for a False Discovery Rate (FDR) of 5%.
